# Evolutionary Analysis of Mitogenomes from Parasitic and Free-Living Flatworms

**DOI:** 10.1371/journal.pone.0120081

**Published:** 2015-03-20

**Authors:** Eduard Solà, Marta Álvarez-Presas, Cristina Frías-López, D. Timothy J. Littlewood, Julio Rozas, Marta Riutort

**Affiliations:** 1 Institut de Recerca de la Biodiversitat and Departament de Genètica, Facultat de Biologia, Universitat de Barcelona, Catalonia, Spain; 2 Department of Life Sciences, Natural History Museum, Cromwell Road, London, United Kingdom; Laboratoire Arago, FRANCE

## Abstract

Mitochondrial genomes (mitogenomes) are useful and relatively accessible sources of molecular data to explore and understand the evolutionary history and relationships of eukaryotic organisms across diverse taxonomic levels. The availability of complete mitogenomes from Platyhelminthes is limited; of the 40 or so published most are from parasitic flatworms (Neodermata). Here, we present the mitogenomes of two free-living flatworms (Tricladida): the complete genome of the freshwater species *Crenobia alpina* (Planariidae) and a nearly complete genome of the land planarian *Obama* sp. (Geoplanidae). Moreover, we have reanotated the published mitogenome of the species *Dugesia japonica* (Dugesiidae). This contribution almost doubles the total number of mtDNAs published for Tricladida, a species-rich group including model organisms and economically important invasive species. We took the opportunity to conduct comparative mitogenomic analyses between available free-living and selected parasitic flatworms in order to gain insights into the putative effect of life cycle on nucleotide composition through mutation and natural selection. Unexpectedly, we did not find any molecular hallmark of a selective relaxation in mitogenomes of parasitic flatworms; on the contrary, three out of the four studied free-living triclad mitogenomes exhibit higher A+T content and selective relaxation levels. Additionally, we provide new and valuable molecular data to develop markers for future phylogenetic studies on planariids and geoplanids.

## Introduction

Complete mitochondrial genomes (mitogenomes) provide a diversity of molecular markers suitable to study a variety of biological features, including the effects of different life habits (e.g. [1]) or the phylogenetic relationships among populations or species. This is because mitochondrial (mt) DNA does not usually recombine, commonly exhibits neutral evolution, and mt markers have smaller effective population sizes than their nuclear counterparts which result in shorter coalescent times [2,3]. These features make mtDNA to be especially appropriate for either phylogeographical or population genetic studies (e.g. [4]).

**Fig 1:**
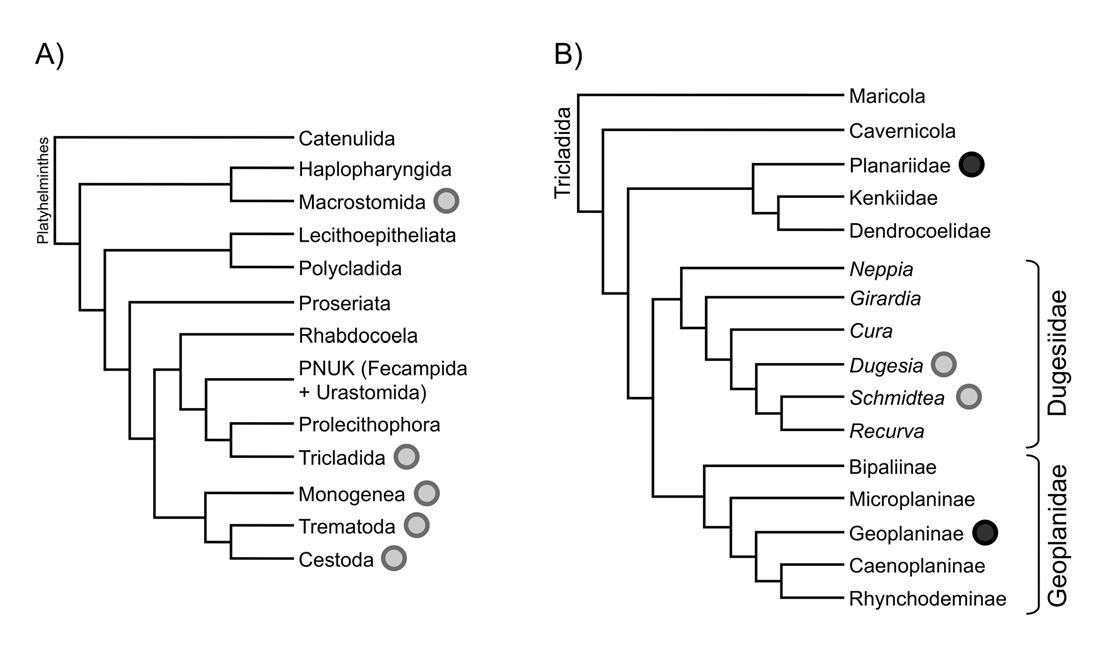
Phylogenetic schemes indicating the relationships of groups for which mitogenomes are available. A) Phylogeny of the Platyhelminthes according to Riutort *et al*., 2012 ([9]) and B) phylogeny of the Tricladida according to Riutort *et al*., 2012 and Sluys *et al*., 2013 ([52]). Monogenea, Trematoda and Cestoda constitute the Neodermata (parasitic flatworms) group. Grey circles indicate those groups for which mitogenomes are already available. Black circles indicate new obtained mitogenomes.

Currently, within the phylum Platyhelminthes (Lophotrochozoa) there is available mitogenome sequence information for up to 40 parasitic species of Neodermata, which includes the Trematoda, Cestoda and Monogenea [5,6]. In contrast, there are few available complete mitogenomes from free-living flatworms [7,8]: one complete mitogenome (*Dugesia japonica*; ~18 kb), another almost complete (*Dugesia ryukyuensis*; ~17 kb) and a fragment of 6.8 kb (*Microstomum lineare*), and also a complete mitogenome of *Schmidtea mediterranea* available in GenBank (Acc. N.: NC_022448.1). Three of these mitogenomes belong to the Tricladida (*Dugesia* and *Schmidtea*), a clade not distantly related to the parasitic flatworms (Fig. 1), although the two groups split possibly in the Paleozoic [9].

The free-living triclads (Tricladida) have been included recently in biogeographical, phylogeographical and conservation studies [10,11]. In particular land planarians have become convenient models for understanding the origins and maintenance of biological diversity because of their low vagility and extreme dependence on the continuity and stability of their habitats. To date, all these studies have been based on partial gene fragments (particularly *cox1*), due to limitations in amplifying other mitochondrial genes or regions.

Through denser taxon sampling the development of universal and specific primers within this group should be achievable. Additionally, this will provide gene order, nucleotide and amino acid data for phylogenetic studies across the phylum, confirming for example the use of the rhabditophoran mitochondrial genetic code for the whole group [12], the identity of initiation and stop codons, and composition skews. Finally, it will also allow a comparison between mitogenomes from free-living and parasitic taxa, providing insights as to whether these different lifestyles have left a molecular signature.

Here we have determined the mitochondrial genomes of two Tricladida species belonging to two different superfamilies (*Crenobia alpina*, Planarioidea; *Obama* sp., Geoplanoidea) with two major aims, (i) to study the molecular evolution of mitochondrial molecules in the platyhelminths and (ii) to determine the putative different impact of natural selection in free-living and parasitic species caused by their lifestyles. In order to achieve the first objective we have compared the sequence and gene annotations of the new mitogenomes together with those of available free-living species (*Dugesia*, [8]; *Schmidtea mediterranea*, Ross *et al*., Acc. N.:NC_022448.1). For the second objective, we used complete mitogenomic data to determine whether parasitic species exhibit higher evolutionary rates or a relaxation of natural selection as previously proposed [13–16]. For the study, we contrasted the impact of mutational and selective strengths on nucleotide composition and codon bias. Additionally, our new mitogenomic data will be useful to further conduct phylogenetic and phylogeographic-based analyses in triclads.

## Material and Methods

### Samples

None of the species used in this study are protected or endangered, and most sampling sites did not require permission for collecting. For *D*. *subtentaculata* locality in Sta. Fe del Montseny within the Parc Natural del Montseny, permission was provided by the Parc authorities. Four species of Tricladida from three different families (Dugesiidae, Geoplanidae, Planariidae) were targeted for complete mitochondrial genome characterization (Table 1). Live specimens of *Crenobia alpina* (Dana, 1766), *Polycelis felina* (Dalyell, 1844), *Dugesia subtentaculata* (Draparnaud, 1801) and *Obama* sp. (*Obama* sp. [17]) were collected from different localities within Catalonia. Sample locality data is shown in Table A in S1 Tables file. It was not possible to obtain the complete mitogenome for two of these species, owing to different reasons, hence the analyses and results from here on will only refer to the species *Crenobia alpina* and *Obama* sp. Information on the problems found and results obtained for the other two species can be found in the S1 File. The complete mitochondrial genomes of two triclads and eight neodermatans were also retrieved from GenBank (Table 1) to carry out a preliminary gene checking of the mitogenomes obtained in this study by means of 454 (Roche) pyrosequencing, and to perform analytical comparisons between triclads and parasitic flatworms.

**Table 1:** List of all Platyhelminthes species included in the present work.

Species	Classification	Life cycle	Acc. Number	Analysis			References
				CG	PGS	SQ	
*Crenobia alpina*	Tricladida, Planariidae	FL	KP208776	X		X	This work
*Dugesia japonica*	Tricladida, Dugesiidae	FL	AB618487.1	X			[8]
*Obama* sp.	Tricladida, Geoplanidae	FL	KP208777	X		X	This work
*Schmidtea mediterranea*	Tricladida, Dugesiidae	FL	NC_022448.1	X			Not published
*Benedenia hoshinai*	Monogenea, Capsalidae	P	NC_014591.1	X			[53]
*Diplogonoporus balaenopterae*	Cestoda, Diphyllobothriidae	P	NC_017613.1	X			[54]
*Fasciola hepatica*	Trematoda, Fasciolidae	P	NC_002546.1	X	X		[23]
*Schistosoma japonicum*	Trematoda, Schistosomatidae	P	NC_002544.1	X			[23]
*Taenia saginata*	Cestoda, Taeniidae	P	NC_009938.1	X			[55]
*Taenia solium*	Cestoda, Taeniidae	P	AB086256.1		X		[21]
*Tetrancistrum sigani*	Monogenea, Ancyrocephalidae s.l.	P	NC_018031.1	X			[56]
*Gyrodactylus derjavinoides*	Monogenea, Gyrodactylidae	P	NC_010976.1	X			[22]

Acronyms indicating the different analyses: CG, Comparative genomics; PGS, Preliminary gene screening; SQ, Sequencing.

Acronyms indicating life cycle: FL, Free-living; P, Parasitic.

### Mitochondrial DNA extraction

We isolated mitochondrial DNA from about 100 animals for each species based on a modification of the protocol described in Bessho *et al*. (1997) [18]. We first removed the mucus from the planarians with a diluted cysteine chloride solution (pH 7.0) obtained from effervescent tablets (CINFA) and then dipped the animals in buffer 1 (0.1 M sucrose, 10 mM TrisHCl, pH 7.4) overnight at −80°C. Animals were next homogenized, transferred to two PPCO tubes and centrifuged at 600 g (Beckman JA-20 rotor) at 2°C during 10 minutes in order to remove nuclei. The supernatant was centrifuged in FEP tubes at 15,000 g at 2°C for 10 minutes in a Sorvall centrifuge (SS-34 rotor). The pellet was dissolved in 40 mL (20 mL in each tube) of 0.1 M sucrose solution containing 50 mM MgCl_2_ (buffer 2). To remove any contamination of nuclear DNA from mitochondrial membranes, the solution was treated with 10 μl of 70 units/mL DNase. After inactivating the DNase (80°C for 10 minutes), 200 mL (100 mL per tube) of 0.6% SDS, 10 mM EDTA, 10 mM Tris-HCl (pH 8.0) (buffer 3) were added and incubated at 60°C for 10 minutes to disrupt mitochondrial membranes. Finally, an ordinary phenol chloroform extraction was applied to isolate mitochondrial DNA [19].

### Mitochondrial DNA quantification and 454 sequencing

We quantified the DNA amount with a Qubit 2.0 fluorometer (Invitrogen) following manufacturer’s instructions. After precipitating the DNA it was resuspended in TE to a final concentration of 20 ng/μL. The five DNA samples were multiplexed identifier (MID) tagged, and the 454 libraries prepared at the Centres Científics i Tecnològics de la Universitat de Barcelona (CCiTUB). The samples were run into a ¼ 454 plate of the GS FLX titanium platform.

### Sequencing reads processing

DNA sequences (reads) and quality information were extracted independently of each MID's in fasta format from the Standard Flowgram Format file (SFF) using the sffinfo script from Roche's Newbler package (454 SFF Tools). We removed adapters, putative contaminant sequences (upon the UniVecdatabase and the *E*. *coli* genome sequence) and reads shorter than 50 bp were removed using the SeqClean (http://compbio.dfci.harvard.edu/tgi/software/) script. All reads with a mean quality score below 20 were trimmed, and the low-quality bases at the ends of the reads were also removed using PRINSEQ [20].

### Sequencing reads post-processing

We determined whether the mitochondrial genes were present in sequencing reads by a BLAST analysis (v. 2.2.24) using available mitochondrial genome data (downloaded from NCBI) of parasitic flatworms (Table 1) as query. In particular we used the protein information of *Taenia solium* [21], *Gyrodactylus derjavinoides* [22] and *Fasciola hepatica* [23] (Table B in S1 Tables file). For the analyses we applied the tBLASTn algorithm (e-value cut-off: 10^-3^), using translation table 9 (echinoderm and flatworm mitochondrial code) to translate DNA information of the 454 reads in all six reading frames.

### Mitochondrial genomes assembling, annotation, PCR amplification and re-sequencing

We first tried to assemble the DNA genome sequence using Newbler 2.6 (454 life Sciences, with settings:-urt-ml 40-mi 85-minlen 50), but with little success. Several short contigs, with a N50 length of about 400 nucleotides, were resolved. However, SeqMan software (DNASTAR, http://www.DNASTAR.com) resolved large nearly complete mtDNA sequences including all filtered 454. The assembled mitogenomes were annotated with Geneious Pro 6.1.7 [24]. Later, we validated the genome assemblies by further Sanger DNA sequencing. This experimental approach allowed us to determine the existence of, and thereby correct, some 454-induced sequence errors (e.g. frameshifts; [25]), to complete the molecules, and to confirm the gene order resulting from the assembled genomes. For such analysis, we designed 34 primers for PCR amplification in *C*. *alpina* and 20 primers for *Obama* sp. (Tables C and D in S1 Tables file) covering the whole length of the genomes. PCR reactions initially included: 1 μl of DNA, 5 μl of Promega 5X Buffer, 1 μl of dNTPs (10 mM), 0.5 μl of each primer (25 μM), 2 μl of MgCl_2_ (25 mM), 0.15 μl of *Taq* polymerase (GoTaq Flexi DNA Polymerase, Promega). Double-distilled and autoclaved water was added to obtain a final 25 μl PCR volume for all molecules. In many cases PCR needed to be optimised by varying annealing temperatures or the concentrations of MgCl_2_ or DNA. PCR products of low yield for direct sequencing were cloned using TOPO TA Cloning Kit of (Invitrogen) following manufacturers' instructions. For every PCR product cloned, five bacterial colonies on average were picked and sequenced in order to obtain representation of the different haplotypes. Cloned fragments were amplified using universal vector primers T3 and T7. All PCR amplicons were purified using the purification kit illustra (GFX PCR DNA and Gel Band of GE Healthcare) or by using a vacuum system (MultiScreen_HTS_ Vacuum Manifold, Millipore). Sequencing reactions, using Big-Dye (3.1, Applied Biosystems) with the same primers used to amplify the fragment, were run on an automated sequencer ABI Prism 3730 (Unitat de Genòmica of Centres Científics i Tecnològics de la Universitat de Barcelona − CCiTUB) or at Macrogen Corporation (Amsterdam, the Netherlands). The chromatograms were visually checked. These additional DNA sequences were aligned and compared with the 454-based assemblies using the software Geneious 6.1.7, which was also used to obtain the final assemblies.

### Prediction of protein-coding genes and rRNA genes

We determined the location of the protein-coding, rrnL and rrnS genes by using a combination of BLAST searches, ORF finder and the Glimmer plug-in in Geneious 6.1.7, MITOS online software [26], and using information from published Platyhelminthes sequences.

We used the online software GenDecoder v1.6 [27] in order to assign the genetic code of the triclads analyzed. As the expected code we used the Echinoderm and Flatworm Mitochondrial Code (translation table 9). We tested all different degrees of Shannon entropy available in the program and we let the removal of columns at 20% of gaps, as it is set as default. We compared our mitogenomes with the Metazoa reference data set, which also includes parasitic platihelmints.

### Prediction of tRNAs

Putative tRNA genes were identified using a combination of the following software: ARWEN (http://130.235.46.10/ARWEN) [28], tRNAscan-SE 1.21 [29], MITOS [26] and DOGMA [30]. The tRNAs not found with these programs were found and annotated by eye with reference to known platyhelminth sequences. In addition to our mtDNA molecules, we included the published *D*. *japonica* mitochondrial genome [8] to double-check the annotation of the molecule.

### Nucleotide composition bias analyses

Comparative analyses of nucleotide composition bias across species or among DNA regions is a powerful approach to determine the impact of mutational and selective pressures on genome evolution. In addition to the standard A+T (or G+C) content, we also estimated the putative nucleotide frequencies bias (NB statistic) from a single strand (the coding strand). Following Shields *et al*. (1988) [31], we defined the NB statistic as:

$$NB=\left[\overset{4}{\underset{i=1}{\sum }}{\left(O_{i}-E_{i}\right)}^{2}/E_{i}\right]/n$$

Where *O*_*i*_ and *E*_*i*_ are the observed and the expected (under equifrequency) numbers of nucleotide variant *i* (*i* = 1, 2, 3, and 4 correspond to A, C, G, and T), and *n* is the total number of positions analyzed. We applied the NB statistic in different portions of the mitochondrial molecule: NBp, NB at the protein coding regions; NB2, NB at the second position of codons; NB3, NB at the third position of four-fold degenerate codons; NBr and NBt, NB at the ribosomal and tRNA genes, respectively.

We also estimated the particular AT and GC strand skews, using the Perna and Kocher (1995) [32] indices, where the AT skew (sAT) is computed as (A−T)/(A+T) and the GC skew (sGC) = (G−C)/(G+C); in both cases the nucleotide frequencies are those of the coding strand. These values range from −1 to +1, where a value of zero indicates that the frequency of A is equal to T (AT skew), or G equal to C (GC skew). We calculated these indices for each gene and for the whole mitochondrial genome of *C*. *alpina* and *Obama* sp., but also for other free-living flatworms with available mitochondrial genome sequence data, and for six selected parasitic species (Table 1). We also computed the sAT (and sGC index) in different functional regions of the mitochondrial molecule, being sATp, the sAT at the protein coding regions; sAT2, sAT at the second position of codons; sAT3, the sAT at the third position of four-fold degenerate codons; sATr and sATt, sAT at the ribosomal and tRNA genes, respectively.

### Codon bias analyses

Analyses of codon bias offer an effective means of disentangling the effects of mutational and selective factors. We estimated the codon usage bias applying the scaled chi-squared (SC) [31], which is a measure based on the chi-square statistic normalized by the number of codons, and Effective Number of Codons statistics (ENC) [33]. For the SC calculation we conducted two types of analyses: for one we used as the expected values those values assuming codon equifrequency (the standard way to compute SC), for the other, we used the observed nucleotide frequencies to determine the expected codon frequency values. For the latter we conducted the analysis separately for each species, and using 4 different types of observed nucleotide frequencies: the SC statistic computed (SCp) using as the expected number of codons (at each codon class) those values based on the observed nucleotide frequency at the protein coding region (the average for all genes within a species); SC2, the SC using information of the observed nucleotide frequencies at the second position of codons; SC3, SC using information at the third position of four-fold degenerate codons; and SCr and SCt, those SC values using the observed nucleotide frequencies at the ribosomal and tRNA genes, respectively.

## Results

### 454 raw data processing, assembling and gene annotation

**Fig 2:**
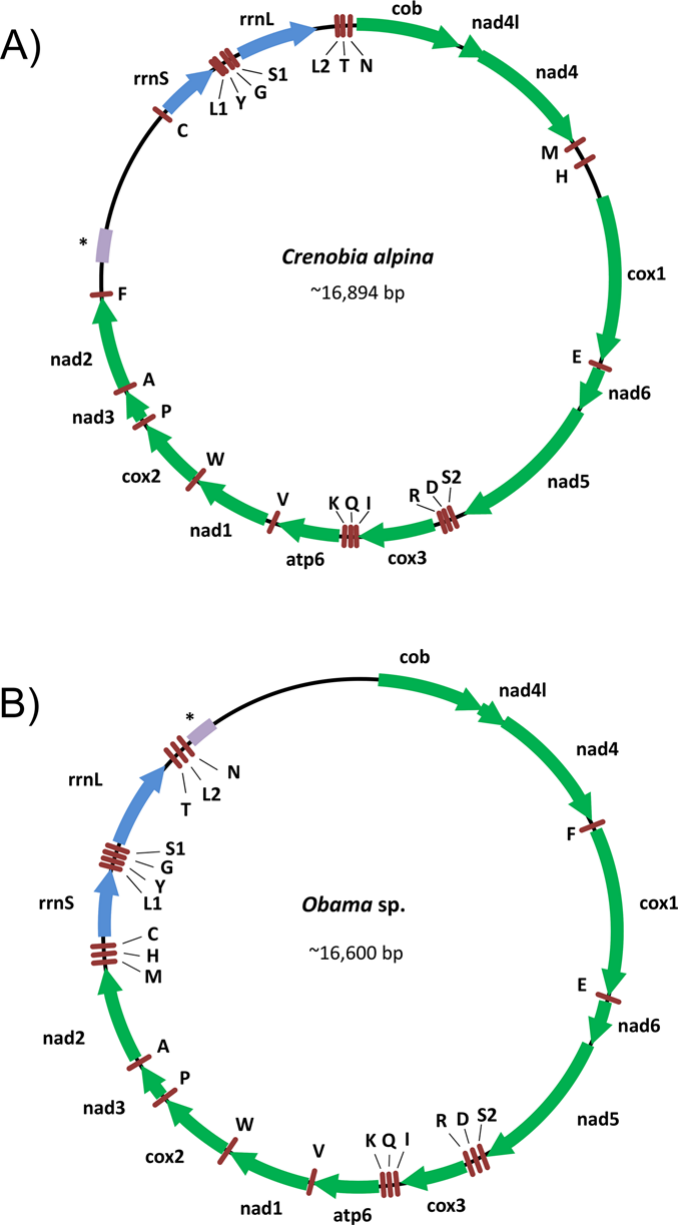
New freshwater flatworm mitogenomes obtained. Arrangement of the mitogenomes of *Crenobia alpina* (A) and *Obama* sp. (B). Green arrows correspond to the protein coding genes; blue arrows ribosomal genes; brown rods tRNAs; Purple bar indicates the putative repetitive region; * indicates that the relative length of the region may be different. Gene identifier: *rrnS*/*rrnL* = small and large subunit rRNA, *nad1–6*,*4L* = NADH dehydrogenase subunits 1–6 + 4L, *cox1–3* = cytochrome c oxidase subunits 1–3, *atp6* = ATP synthase subunit 6, *cob* = cytochrome b. The tRNAs are shown according to the amino acid code letter.

The summary statistics for the 454 sequencing are shown in Table E in S1 Tables file. The 454 reads of *C*. *alpina* and *Obama* sp. provided sufficient information to assemble the mitogenomes successfully (Fig. 2 and Table F in S1 Tables) while it was not possible for the other three species (see S1 File). The SeqMan assembly of *C*. *alpina* generated a single contig of 17,079 bp. The average coverage of the assembly was 29.1X. For *Obama* sp. we obtained a contig of 14,893 bp with an average coverage of 24.3X. In this case, the quality of the DNA sequence was poorer than that obtained for *C*. *alpina*, likely by an increased 454 error rate in *Obama* sp. caused by a higher frequency of homo-polymer sequences. Both assemblies included all mitochondrial genes but lacked a large portion of the non-coding regions.

We completed and checked the sequences of these preliminary assemblies by Sanger DNA sequencing. We carried out additional partial PCR amplifications on the basis of the first assembly, and identified missing and/or extra bases. For instance, in the first assembly of *C*. *alpina* there was a missing nucleotide (a 454 error) in *nad4* and *nad5*, leading to a putative (erroneous) frameshift. This situation also occurred in several genes of the *Obama* sp. assembly.

It was not possible to re-sequence by Sanger the complete mitogenome of *C*. *alpina* since the designed primers failed to PCR amplify a fragment containing a repetitive region of about 186 bp (consensus size) (Fig. 2A). Indeed, the 454 assembly of this region recovered only two copies of this repetitive sequence likely due to the limitation of 454 read lengths. However, when the 454 reads were mapped to the whole mitochondrial molecule this region showed much higher sequence coverage than the rest of. the molecule suggesting that there were more than two repeat units, likely around four. Hence we do not know the exact number of repeats present in this region, and thus the total length of the full mitogenome.

For *Obama* sp. we PCR amplified a band of around 2,000 bp from the 3’ end of *rrnL* to the 5’ end of *cob* gene. However, it was not possible to obtain clean Sanger sequences probably due to the presence of a repetitive region within this fragment (Fig. 2B), hence the complete mitogenome length is also unknown for this species.

The mitochondrial genome of *C*. *alpina* (estimated size >16,894 bp; GenBank ID: KP208776) and *Obama* sp. (estimated size ~16,600 bp; GenBank ID: KP208777) encode 12 protein-coding genes, 22 tRNA genes and 2 ribosomal genes (Fig. 2 and Tables G and H in S1 Tables file), all transcribed from the same strand. As with other platyhelminths *nad4l* gene was the single case of one protein coding gene overlapping another; in *Obama* sp. and *C*. *alpina nad4l* overlaps 32 bp with *nad4*. In *Obama* sp., there may be (i) an overlap of 17 bp with *cob*, or (ii) no overlap and an alternative stop codon for *cob* one nucleotide before the start of *nad4l* (a codon presenting two ambiguous positions: TWW).

GenDecoder results support the use of the Echinoderm and Flatworm Mitochondrial Code for *Obama* sp. and *Crenobia alpina*. We found differences between the expected and predicted translation for some codons; one or two for *Obama* sp. and one to five for *Crenobia alpina* depending on the degree of Shannon entropy. However, these alternative translations were weakly supported, considered as unreliable predictions, thus supporting our expected code.

### Gene order

The protein coding gene (PCG) order is conserved across Tricladida, but it is radically different from the incomplete fragment available from another free-living flatworm, *Microstomum*, and all the parasitic species (S1 Fig). Only three blocks of genes are conserved between parasites and triclads (S2 Fig). Our re-annotation of the *D*. *japonica* mitogenome entailed the change of three tRNAs to positions more similar, or identical, to those found in the other triclads: *trnC* is on the same strand as the rest of genes and *trnA* and *trnL1* are in the same relative position than in the other triclads (S3 Fig). In spite of these corrections all four triclad species (*C*. *alpina*, *Obama* sp., *S*. *mediterranea* and *D*. *japonica*) exhibit differences in the location of some tRNAs (S4 Fig).

The ribosomal genes are located close to the long non-coding region in the four Tricladida species, although in a different position. For *C*. *alpina* and *S*. *mediterranea* the long non-coding region is situated 5’ upstream of the ribosomal genes while for *Obama* sp., and *D*. *japonica* it is situated at its 3’ end. In contrast to other platyhelminth mitogenomes *rrnS* is situated upstream of *rrnL* amongst triclads (S1 Fig).

### Start and terminal codons

We infer that four start codons are used in the two species analyzed. TTG and ATG are used at equivalent frequencies in *Obama* sp. while ATG is more frequent than TTG in *C*. *alpina*, TTA is also used in both species and GTG only in *Obama* sp. (Tables G and H in S1 Tables file). Stop codons are TAG and TAA. In *C*. *alpina*, *cox2* gene has a TAR stop codon, showing the presence of the two possible stop codons within the population (heterozygosity). Alternatively this could be a case of a truncated TA stop codon.

The length of the genes is very similar between the two species. However, in general the predictions for *Obama* sp. are slightly longer resulting in a more compact genome (shorter intergenic regions).

### Transfer RNAs and ribosomal genes

Most tRNA genes in *C*. *alpina* and *Obama* sp. have the classical secondary structure (S5 and S6 Figs). The tRNAs *trnS2* and *trnT* lack the DHU arm in both species, while in *C*. *alpina* the *trnQ* could have two alternative structures: either lacking the TΨC arm or the DHU arm.

In *C*. *alpina*, four tRNAs overlap (*trnI*, *trnW*, *trnA*, *trnF*) with the last two bases of four genes (*cox3*, *nad1*, *nad3*, *nad2* respectively). Moreover, *trnL1* overlaps with *trnY*. In *Obama* sp., *trnF* and *trnV* overlap 1 nucleotide with genes *nad4* and *atp6* respectively. On the other hand, there are 3 cases of overlapping between tRNAs (*trnD* and *trnR*, 5 bp; *trnQ* and *trnK*, 8 bp; *trnY* and *trnG*, 4 bp). In the new annotation of *D*. *japonica* mitogenome the *trnA* and *trnL1* preserve the four arms while *trnC* lacks TΨC arm (S7 Fig).

### Non-coding regions

*C*. *alpina* long non-coding region contains at least four repeats of 186 bp (consensus size) between two non-repetitive regions of 309–311 bp upstream and of 1,363 bp downstream. The total length of this large non-coding region is, at least, 2,028 bp. In the case of *Obama* sp. we only have the information of the length of the amplified fragment, around 2,000 bp, but we cannot establish the true number of repeat elements.

### Nucleotide composition, strand skew and codon usage bias

**Fig 3:**
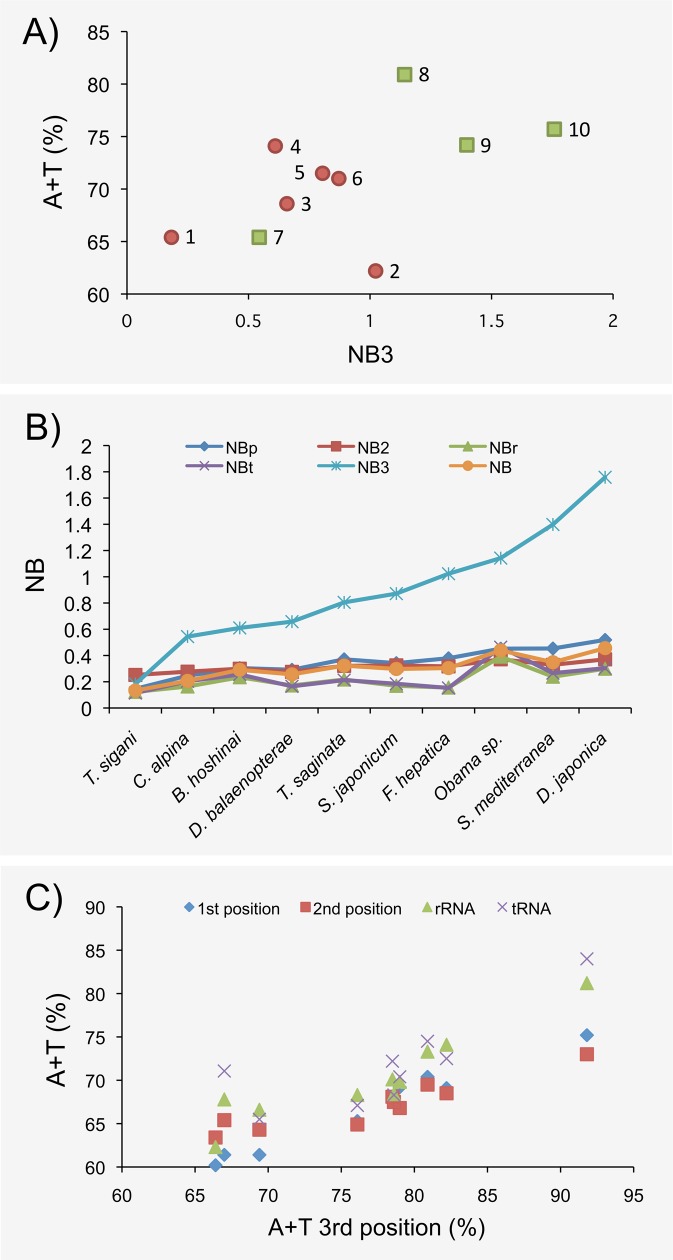
Nucleotide composition bias in the Platyhelminthes analyzed. A) Relationship between A+T content and NB3 (NB at the third position of four-fold degenerate codons) values. Green squares and red circles indicate free-living and parasitic platyhelminths, respectively. The surveyed species are shown in numbers: 1, *T*. *sigani*; 2, *F*. *hepatica*; 3, *D*. *balaenopterae*; 4, *B*. *hoshinai*; 5, *T*. *saginata*; 6, *S*. *japonicum*; 7, *C*. *alpina*; 8, *Obama* sp.; 9, *S*. *mediterranea*; 10, *D*. *japonica*. B) Values of the different NB-based statistic across species. C) Relationship between A+T content for different genome portions and A+T content for the third positions.

Triclad mitogenomes have high A+T content values (>60%) (Fig. 3A). The per strand nucleotide frequency bias is also noticeably high, both in free-living and parasitic species (Fig. 3B; S8 Fig). We found such bias both at the whole molecule (NB statistic) and in different portions of the same (NBp, NB2, NB3, NBr and NBt), with bias at the third position of codons (NB3) being more pronounced. The A+T content at the third position of codons correlates with that frequency in the 1^st^, the 2^nd^, the rRNA and tRNA sites (Fig. 3C). These analyses separate the surveyed species into two clusters, parasitic and free-living species (with the exception of *C*. *alpina*).

**Fig 4:**
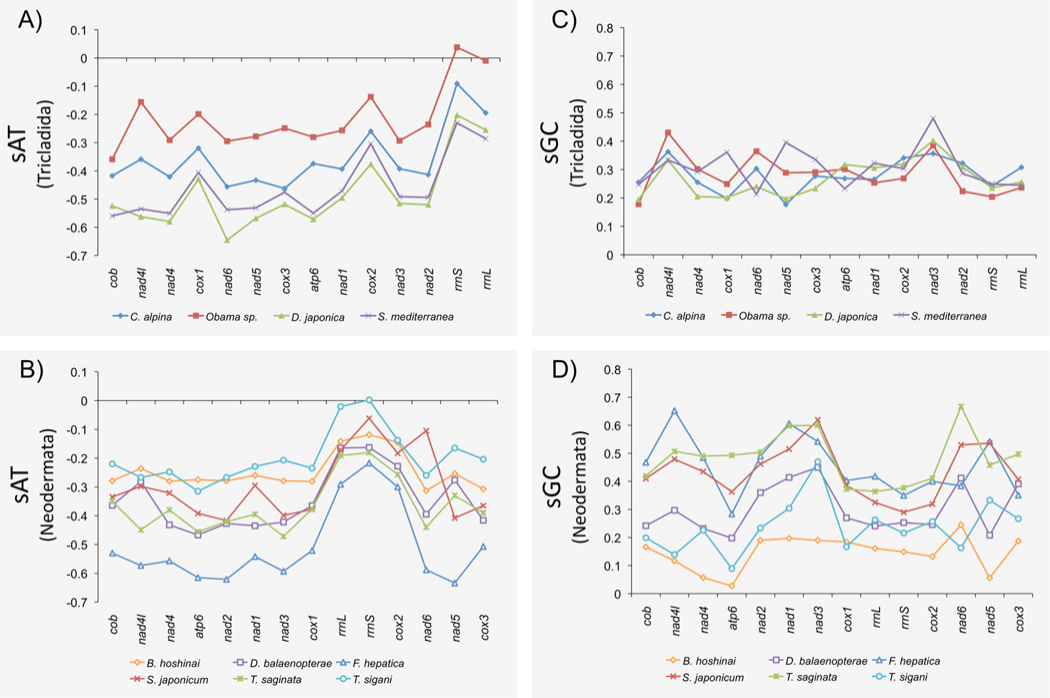
sAT and sGC values of the protein coding genes (PCG) along the mtDNA molecule. A) sAT of Tricladida; B) sAT of Neodermata; C) sGC of Tricladida; D) sGC of Neodermata.

In contrast to the A+T and NB values, free-living and parasitic species do not differentiate themselves from one another with respect to sAT or sGT values, either for the total data or for the values estimated at positions with different functional behavior (S9 and S10 Figs.). All sAT values are negative (in all genes and in all species), with the exception of the *rrnS* gene of *Obama* sp. and *T*. *sigani* where values are slightly positive (Fig. 4A and 4B). Thus, there is a clear prevalence of T over A in the coding strand. Moreover, the general sAT skew varies considerably among species (−0.187 to −0.4 Tricladida; −0.168 to −0.483 Neodermata), but it is consistent across genes; for instance *F*. *hepatica* has the highest overall sAT values, a feature exhibited in all of its genes (Fig. 4B). The sAT and A+T content, however, are uncoupled; for instance, *Obama* sp., the species with highest A+T content, exhibits nearly the lowest sAT values. The general sGC estimates also show important strand skews, ranging from 0.246 to 0.283 in triclads and 0.148 to 0.475 in parasites, which indicate a higher frequency of G than C. Although the sGC values also show some species-specific pattern it is much less consistent across genes. Overall, the analyses uncover a species-specific pattern that (i) is not correlated with the actual A+T content (S9 Fig), (ii) differs between sGC and sAT estimates, and (iii) does not cluster free-living or parasitic species separately.

**Fig 5:**
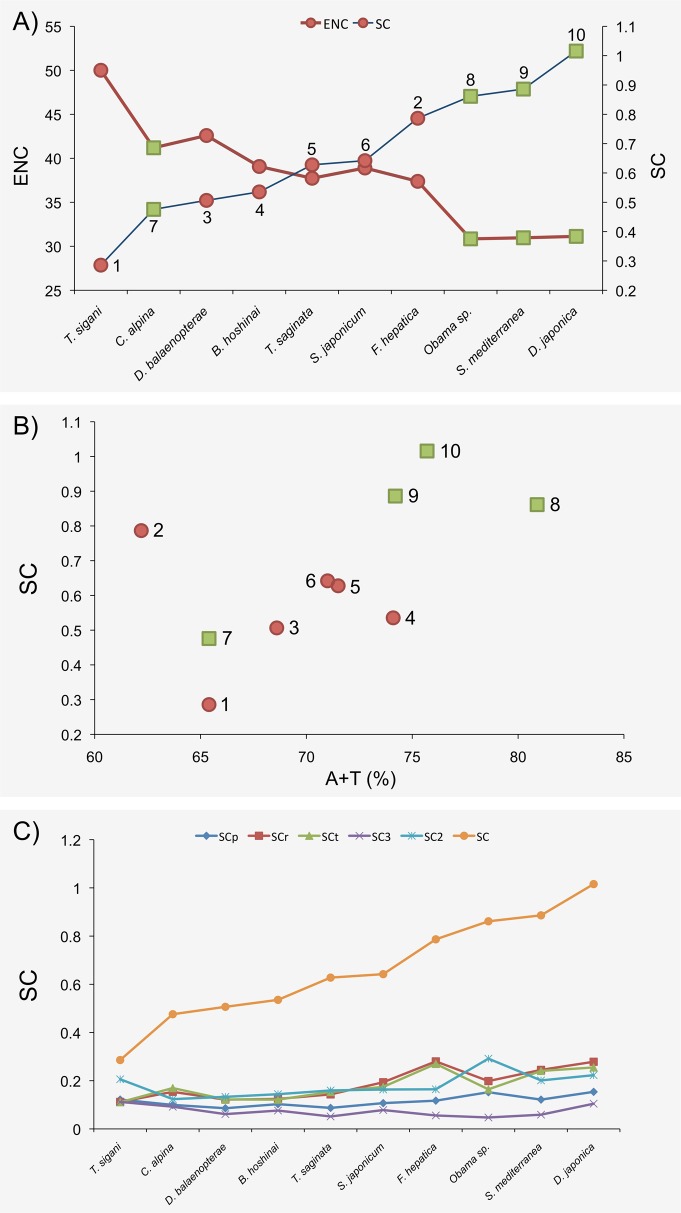
Relationship between different codon bias measures. A) Relationship between ENC and SC values. B) Relationship between SC and A+T% values. C) SC values across species (see Material and Methods text for acronym description). Green squares and red circles indicate free-living and parasitic Platyhelminthes, respectively. The surveyed species are shown in numbers: 1, *T*. *sigani*; 2, *F*. *hepatica*; 3, *D*. *balaenopterae*; 4, *B*. *hoshinai*; 5, *T*. *saginata*; 6, *S*. *japonicum*; 7, *C*. *alpina*; 8, *Obama* sp.; 9, *S*. *mediterranea*; 10, *D*. *japonica*.

The results of the codon usage analysis also show high levels of bias across the surveyed species (Fig. 5A and 5B), both using the SC or ENC estimators. Interestingly, and in agreement with the nucleotide frequency bias analyses, the free-living species again show the highest levels of codon bias (excepting *C*. *alpina*).

## Discussion

### Mitogenomes of Tricladida: general features

The mitogenomes of the newly characterized triclad species, *Crenobia alpina* and *Obama* sp., share the same gene composition with the majority of the Platyhelminthes sequenced so far, 12 PCGs while the *atp8* gene is absent. This gene is also absent in the mitogenomes of Chaetognatha, and Rotifera among lophotrochozoans as well as in some Bivalvia (Mollusca) and most Nematoda [6,34,35]. They also encode for the usual complement of 22 tRNAs, as found in almost all other platyhelminth genomes; two species of the digenean genus *Schistosoma* (*S*. *japonicum* and *S*. *mansoni*) have 23 due to a duplication of the *trnC* gene [6]. Also, all genes are transcribed from the same strand, a feature found in other Platyhelminthes, Cnidaria, Porifera, Tunicata and many other lophotrochozoan phyla [6,34].

The genetic code used by all triclad species is consistent with that used for the majority of Platyhelminthes, i.e., the EMBL-NCBI genetic code 9: Echinoderm and Flatworm. We found no evidence that codon TAA codes for Tyr (as proposed by Bessho *et al*. 1992 [36]); on the contrary TAA appears to be the stop codon for most of our predicted genes, and in some of *D*. *japonica* [8]. Hence the “alternative flatworm mitochondrial code”, code 14 from EMBL-NCBI, proposed for some Platyhelminthes [36] and Nematoda is likely a feature exclusive to the latter.

### Gene order

The PCG order is identical in *C*. *alpina* and *Obama* sp. (Figs. 2 and S3), and also with the mitochondrial genomes of *D*. *japonica*, *D*. *ryukyuensis* and *S*. *mediterranea*. The only differences include the identity and arrangement of the tRNAs and the relative position of the long non-coding regions. The similarity in the situation of the non-coding region between *C*. *alpina* and *S*. *mediterranea* is surprising considering the closer phylogenetic relationships between *S*. *mediterranea* and *Dugesia* and *Obama*, all belonging to the superfamily Geoplanoidea, sister to the Planarioidea to which *Crenobia* belongs (Fig. 1B). On the other hand, the small number of changes in tRNAs order (S4 Fig) among all Tricladida is a notable feature given the very likely antiquity of the lineage.

The gene order among Tricladida differs considerably from that found in the parasitic platyhelminths and in *Microstomum*. One unique feature for Tricladida is the relative position of the two ribosomal genes; *rrnS* is located at 5' from *rrnL*, being the other way around in all the other platyhelminth mitogenomes characterized to date. Futhermore, in neodermatans *rrnL* and *rrnS* are flanked by *cox1* and *cox2*, whereas in triclads *rrnS* and *rrnL* are flanked by *nad2* and *cob*.

### Start and terminal codon usage

While parasitic flatworms use only ATG and GTG as start codons, with the exception of a GTT used in *Hymenolepis diminuta* [6,37], Tricladida (Tables G, H and I in S1 Tables file; [8]) have much higher versatility. In addition to ATG and GTG, this group also appears to use TTG as start codon, and perhaps TTA and TAT. Moreover, the start codon for each gene is not conserved across Tricladida; in fact, only the start codon of *atp6* (TTG) is shared between all triclads. This diversity suggests independent origins of such codons across species. Although abbreviated stop codons (TA or T) are common in animal mitogenomes ([38] and references therein), we found that triclads have standard trinucleotide stop codons. In *Obama* sp., 10 out of the 12 PCG terminate in TAA, while *D*. *japonica* has the reverse situation 10 out of 12 PCG have TAG as stop codon. In *C*. *alpina* and *S*. *mediterranea* the usage of both stop codons is almost the same. The preference of the TAA stop codons in *Obama* sp. could be explained by the high frequency of A over G along its genome. The situation in the other three species with a similar proportion of A and G can explain the proportions of stop codons found in *S*. *mediterranea* and *C*. *alpina*, but not in *D*. *japonica*.

Although we used different methods to infer the start and stop codons for each gene, the lack of transcriptional information precludes any interpretation of boundaries with a high degree of confidence. Future studies involving transcriptomic analyses will help for a more accurate annotation of these species' genes.

### A+T content and asymmetric strand bias

We have found that triclads have high A+T content values, a feature already detected in parasitic flatworms. Nevertheless, while some parasitic species have A+T content values around 70%, *Obama* sp. exhibits a much more extreme bias (over 80%), close to the highest described cases (Hymenoptera; [39]).

The surveyed triclad species exhibit negative sAT and positive sGC skew values in the coding strand, a typical feature also reported in other Platyhelminthes [6,40]. It has been proposed that this feature would be linked to the replication process [41–43]. That is, the longer strands are kept single during replication, the higher the likelihood of depurination mutations resulting in substitutions from A to G and from C to T (100 times more frequent). However, analysis of the sAT and sGC levels in the PCG as a function of their relative physical order does not show the predicted pattern; instead, there is a clear species-specific signature with contrasting values across species (Fig. 4). The fact that the A+T content (or the NB3 value) and skew values do not correlate across species (S9 and S10 Figs.) does not support the mutational input as a major source for the skew. The situation is the same when we consider the skews for only second or third sites within the coding regions (S9B and S9C Fig; S10B and S10C Fig). These results suggest that the asymmetric nucleotide composition strand bias has some significance, a feature that could be related to the fact that all genes are located on the same strand (see [44]).

### Effect of natural selection on free-living and parasitic species

It has been proposed that parasitic species might exhibit a relaxation of natural selection, as compared with free-living organisms, because of a putative reduction in their effective population sizes [45,46]. Changes in the selection regime may imprint a plethora of characteristic molecular hallmarks on DNA and protein sequences that eventually can be detected. For instance, the relaxation of the intensity of natural selection can cause an increase of the nucleotide and amino acid substitution rates, a decrease in the selective constraint levels (increased values of ω = *d*_N_/*d*_S_ parameter), and an increase in the mutational bias. The effect of such relaxation on the codon usage bias, however, is likely to be more complex: a reduction of codon bias if the bias is actively maintained by natural selection, but an increase if mutation is the stronger force [47]. Here we have taken advantage of the availability of complete mtDNA data for a number of flatworm species to gain insights into this issue. Unfortunately, we cannot analyze either the putative different patterns left on the evolutionary rates (there is no reliable data of divergence times) or its impact of selective constraint levels because of the high saturation of *d*_S_ values.

The high A+T content value in all species analyzed, as expected, produces a substantial nucleotide frequency bias. Interestingly, the more pronounced bias corresponds to the NB3 statistic (Fig. 3B), where the highest biases are in species exhibiting the highest A+T content values (Fig. 3A). This result points to mutation, and not to natural selection, as the major evolutionary force responsible for the bias in the nucleotide frequencies. It can be argued that the high levels of A+T may be in fact driven by natural selection acting on the third positions of codons (to get a more efficient codon usage). Nevertheless, we can reject the selective hypothesis since the correlation of the A+T frequency with the frequencies at third codon positions is also observed at the 1^st^, the 2^nd^, the rRNA and tRNA sites (Fig. 3C). Remarkably, the free-living and parasitic species differ considerably in their nucleotide frequency bias, with free-living species having higher values (with the exception of *C*. *alpina*). Moreover, this pattern is consistent across the different NB measures (S8 Fig).

Interestingly, the pattern of codon usage bias reflects that shown by the nucleotide frequency analyses. The codon bias might be a by-product of the mutational input or might result from the action of natural selection for increased translational efficiency or accuracy [48–51]. To disentangle both effects we studied the level of codon bias adjusting for the observed mutation bias (Fig. 5C; S11 Fig). As expected if codon bias mainly results from some form of mutational bias, the SC values drop dramatically, and especially for SC3 values. However, we do not observe any clear pattern that differentiates free-living from parasitic species. Moreover, using different SC-mutational adjusting estimators yields different species-rank orders and, therefore, the separate clustering of free-living (except *C*. *alpina*) from parasites species on basis of their SC values disappears.

Our results on the impact of nucleotide and codon bias indicate that parasitic platyhelminth species do not exhibit a higher relaxation of natural selection than free-living species. On the contrary, three out of the four free-living species (Geoplanoidea representatives) exhibit patterns of A+T content and nucleotide frequency bias in clear agreement of mutation as the major evolutionary driver. Our results further reveal that the observed codon bias is primarily caused by mutation and not by natural selection mechanisms. Likewise, the high diversity of start codons uncovered in these free-living species and their usage of stop codons can also be explained by a putative relaxation of natural selection (see start and terminal codon usage section). Globally these results agree with that found for bacteria [47], although differ from some studies of plants, in which mutation appears to have a higher impact than natural selection in parasitic relative to non-parasitic species [16]. In summary, although it has been proposed that life cycles of parasitic species render them more prone to suffering genetic bottlenecks that in turn may lead to putative reductions on the effective population sizes, we did not find the molecular hallmark of a relaxed selection force in the parasitic Platyhelminthes. On the contrary, free-living triclads appear to exhibit higher levels of relaxed selection. In fact their vagility and requirements for persistent habitats may render these species highly vulnerable, very susceptible to local extinctions and recolonizations, which in turn could explain these results. In any case, our conclusions suggest that the relaxed selection proposed for some parasites is not a general feature of parasitic organisms.

## Supporting Information

S1 Fig Linearized schemes of gene orders in Platyhelminthes. Those genes that are variable within each of the three parasitic groups (Cestoda, Monogenea and Trematoda) are in bold. Multiple genes in the same box indicate variable gene orders within the specific group. Gene identifier as in Fig. 2. The tRNAs are shown according to the amino acid code letter. Gene orders derived from the mt genomes of *Diphyllobothrium latum*, *D*. *nihonkaiense*, *Diplogonoporus balaenopterae*, *D*. *grandis*, *Echinococcus canadensis*, *E*. *equinus*, *E*. *granulosus*, *E*. *multilocularis*, *E*. *oligarthrus*, *E*. *ortleppi*, *E*. *shiquicus*, *E*. *vogeli*, *Hymenolepis diminuta*, *Spirometra erinaceieuropaei*, *Taenia asiatica*, *T*. *crassiceps*, *T*. *hydatigena*, *T*. *multiceps*, *T*. *pisiformis*, *T*. *saginata*, *T*. *solium*, *T*. *taeniaeformis* for CESTODA; *Benedenia hoshinai*, *B*. *seriolae*, *Tetrancistrum nebulosi* for MONOGENEA 1; *Gyrodactylus derjavinoides*, *G*. *salaris*, *G*. *thymalli* for MONOGENEA 2; *Microcotyle sebastis*, *Polylabris halichoeres*, *Pseudochauhanea macrorchis* for MONOGENEA 3; *Clonorchis sinensis*, *Fasciola hepatica*, *Opisthorchis felineus*, *Paragonimus westermani* for TREMATODA 1; *Schistosoma japonicum*, *Sc*. *mekongi*, *Trichobilharzia regent* TREMATODA 2; *Schistosoma haematobium*, *Sc*. *mansoni*, *Sc*. *spindale* for TREMATODA 3. Based on Wey-Fabrizius *et al*., 2013.
(PDF)

S2 Fig Linearized scheme showing a comparison of the general protein coding and ribosomal genes between generalized mitogenomes of Neodermata (parasitic platyhelminths) and Continenticola (land planarians and freshwater triclads; all free-living). (PDF)

S3 Fig Comparison between the annotation proposed by Sakai and Sakaizumi (2012) for *Dugesia japonica* and the new annotation proposed in the present study. (PDF)

S4 Fig Comparison by pairs of the tRNA order of the different Tricladida species included in this work. (PDF)

S5 Fig Secondary structure of the 22 tRNA of *Crenobia alpina*. trnQ* in a box shows the alternative structure proposed for this tRNA. The different tRNA parts are showed on *trnD*.
(PDF)

S6 Fig Secondary structure of the 22 tRNA of *Obama* sp. (PDF)

S7 Fig Comparison between the Sakai and Sakaizumi (2012) *trnA*, *trnC* and *trnL1* secondary structure for *Dugesia japonica* based on their annotation and the secondary structure based on our new proposed annotation. (PDF)

S8 Fig Values of the different NB-based statistic across species excluding the NB3 (NB at the third position of four-fold degenerate codons). (PDF)

S9 Fig Relationship between sAT, sGC values and NB3. sAT general skew; sAT2, sAT skew at the second positions; sAT3, sAT at the third positions. sGC, general skew; sGC2, sGC skew at the second positions; sGC3, sGC at the third positions. Green squares and red circles indicate free-living and parasitic platyhelminths, respectively. The surveyed species are shown in numbers: 1, *T*. *sigani*; 2, *F*. *hepatica*; 3, *D*. *balaenopterae*; 4, *B*. *hoshinai*; 5, *T*. *saginata*; 6, *S*. *japonicum*; 7, *C*. *alpina*; 8, *Obama* sp.; 9, *S*. *mediterranea*; 10, *D*. *japonica*.
(PDF)

S10 Fig Relationship between sAT and sGC values and A+T content. sAT general skew; sAT2, sAT skew at the second positions; sAT3, sAT at the third positions. sGC, general skew; sGC2, sGC skew at the second positions; sGC3, sGC at the third positions. Green squares and red circles indicate free-living and parasitic Platyhelminthes, respectively. The surveyed species are shown in numbers: 1, *T*. *sigani*; 2, *F*. *hepatica*; 3, *D*. *balaenopterae*; 4, *B*. *hoshinai*; 5, *T*. *saginata*; 6, *S*. *japonicum*; 7, *C*. *alpina*; 8, *Obama* sp.; 9, *S*. *mediterranea*; 10, *D*. *japonica*.
(PDF)

S11 Fig SC values across species adjusted for the observed mutation bias. Ordered ascending based on the Chi scales values for A) second positions of the PCG and B) for the third position of four-fold degenerate codons equifrequency.
(PDF)

S1 File Supplementary information on negative results for *Polycelis felina* and *Dugesia subtentaculata*. (DOCX)

S1 Tables Table A. Locality and habitat information on species collected for this study. Table B. Data of mitochondrial proteins used to conduct the tBLASTx analyses in order to detect whether the mitochondrial genes were present in the 454 sequencing reads. Table C. Primers designed for the reamplification of *Crenobia alpina*. Table D. Primers designed for the reamplification of *Obama* sp. Table E. Summary statistics for the 454 sequencing. Table F. Summary of tBLASTn hits for raw reads against the mitochondrial proteins of the three parasitic flatworms. Table G. Annotation table for the mitochondrial genome of *C*. *alpina*. Table H. Annotation table for the mitochondrial genome of *Obama* sp. Table I. Annotation table for the mitochondrial genome of *S*. *mediterranea*.
(DOCX)
