# Supplementary material for: Evolutionary Analysis of Mitogenomes from Parasitic and Free-Living Flatworms
Source: PLoS One. 2015 Mar 20;10(3):e0120081. doi: 10.1371/journal.pone.0120081 (PMC4368550; doi:10.1371/journal.pone.0120081)
Supplement: S1 Fig — Those genes that are variable within each of the three parasitic groups (Cestoda, Monogenea and Trematoda) are in bold. Multiple genes in the same box indicate variable gene orders within the specific group. Gene identifier as in Fig. 2. The tRNAs are shown according to the amino acid code letter. Gene orders derived from the mt genomes of Diphyllobothrium latum, D. nihonkaiense, Diplogonoporus balaenopterae, D. grandis, Echinococcus canadensis, E. equinus, E. granulosus, E. multilocularis, E. oligarthrus, E. ortleppi, E. shiquicus, E. vogeli, Hymenolepis diminuta, Spirometra erinaceieuropaei, Taenia asiatica, T. crassiceps, T. hydatigena, T. multiceps, T. pisiformis, T. saginata, T. solium, T. taeniaeformis for CESTODA; Benedenia hoshinai, B. seriolae, Tetrancistrum nebulosi for MONOGENEA 1; Gyrodactylus derjavinoides, G. salaris, G. thymalli for MONOGENEA 2; Microcotyle sebastis, Polylabris halichoeres, Pseudochauhanea macrorchis for MONOGENEA 3; Clonorchis sinensis, Fasciola hepatica, Opisthorchis felineus, Paragonimus westermani for TREMATODA 1; Schistosoma japonicum, Sc. mekongi, Trichobilharzia regent TREMATODA 2; Schistosoma haematobium, Sc. mansoni, Sc. spindale for TREMATODA 3. Based on Wey-Fabrizius et al., 2013. (PDF) [file pone.0120081.s001.pdf]

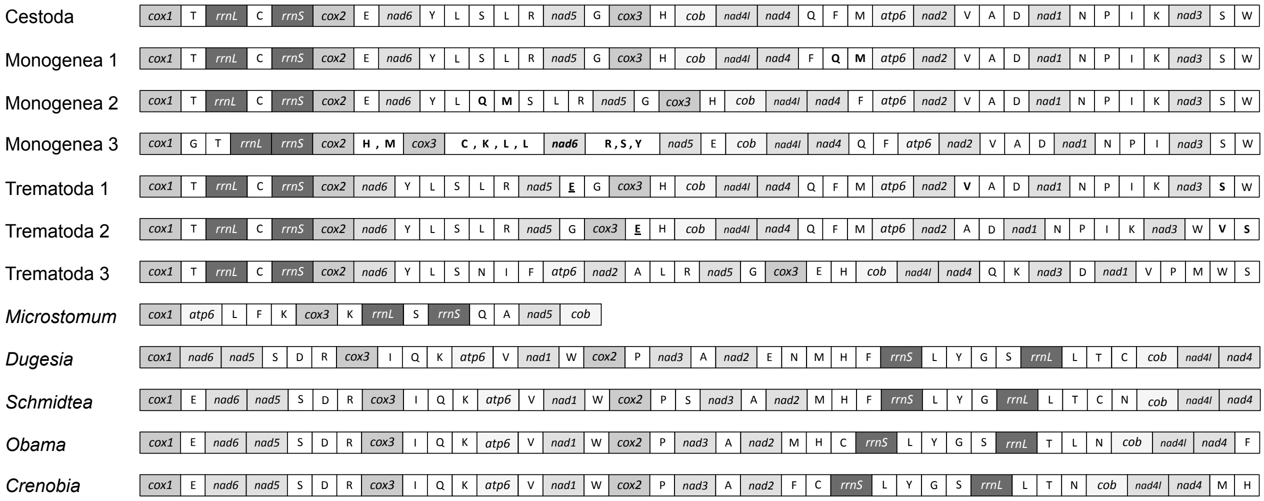
Cestoda cox1 T rrnL C rrnS cox2 E nad6 Y L S L R nad5 G cox3 H cob nad4l nad4 Q F M atp6 nad2 V A D nad1 N P I K nad3 S W
Monogenea 1 cox1 T rrnL C rrnS cox2 E nad6 Y L S L R nad5 G cox3 H cob nad4l nad4 F Q M atp6 nad2 V A D nad1 N P I K nad3 S W
Monogenea 2 cox1 T rrnL C rrnS cox2 E nad6 Y L Q M S L R nad5 G cox3 H cob nad4l nad4 F atp6 nad2 V A D nad1 N P I K nad3 S W
Monogenea 3 cox1 G T rrnL rrnS cox2 H , M cox3 C , K , L , L nad6 R , S , Y nad5 E cob nad4l nad4 Q F atp6 nad2 V A D nad1 N P I nad3 S W
Trematoda 1 cox1 T rrnL C rrnS cox2 nad6 Y L S L R nad5 E G cox3 H cob nad4l nad4 Q F M atp6 nad2 V A D nad1 N P I K nad3 S W
Trematoda 2 cox1 T rrnL C rrnS cox2 nad6 Y L S L R nad5 G cox3 E H cob nad4l nad4 Q F M atp6 nad2 A D nad1 N P I K nad3 W V S
Trematoda 3 cox1 T rrnL C rrnS cox2 nad6 Y L S N I F atp6 nad2 A L R nad5 G cox3 E H cob nad4l nad4 Q K nad3 D nad1 V P M W S
Microstomum cox1 atp6 L F K cox3 K rrnL S rrnS Q A nad5 cob
Dugesia cox1 nad6 nad5 S D R cox3 I Q K atp6 V nad1 W cox2 P nad3 A nad2 E N M H F rrnS L Y G S rrnL L T C cob nad4l nad4
Schmidtea cox1 E nad6 nad5 S D R cox3 I Q K atp6 V nad1 W cox2 P S nad3 A nad2 M H F rrnS L Y G rrnL L T C N cob nad4l nad4
Obama cox1 E nad6 nad5 S D R cox3 I Q K atp6 V nad1 W cox2 P nad3 A nad2 M H C rrnS L Y G S rrnL T L N cob nad4l nad4 F
Crenobia cox1 E nad6 nad5 S D R cox3 I Q K atp6 V nad1 W cox2 P nad3 A nad2 F C rrnS L Y G S rrnL L T N cob nad4l nad4 M H
