# Supplementary material for: Evolutionary Analysis of Mitogenomes from Parasitic and Free-Living Flatworms
Source: PLoS One. 2015 Mar 20;10(3):e0120081. doi: 10.1371/journal.pone.0120081 (PMC4368550; doi:10.1371/journal.pone.0120081)
Supplement: S2 Fig — (PDF) [file pone.0120081.s002.pdf]

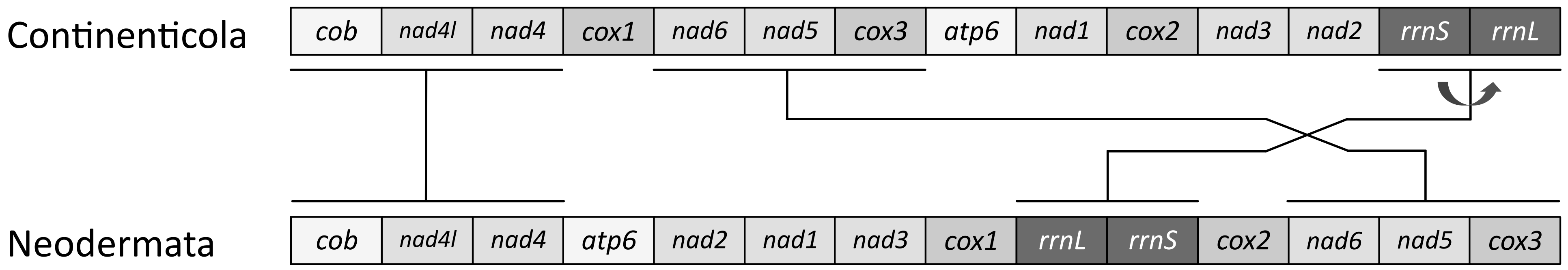

Continenticola
cob
nad4l
nad4
cox1
nad6
nad5
cox3
atp6
nad1
cox2
nad3
nad2
rrnS
rrnL
Neodermata
cob
nad4l
nad4
atp6
nad2
nad1
nad3
cox1
rrnL
rrnS
cox2
nad6
nad5
cox3
