# Supplementary figures and images for: Evolutionary Analysis of Mitogenomes from Parasitic and Free-Living Flatworms
Source: PLoS One. 2015 Mar 20;10(3):e0120081. doi: 10.1371/journal.pone.0120081 (PMC4368550; doi:10.1371/journal.pone.0120081)

Sakai and Sakaizumi, 2012

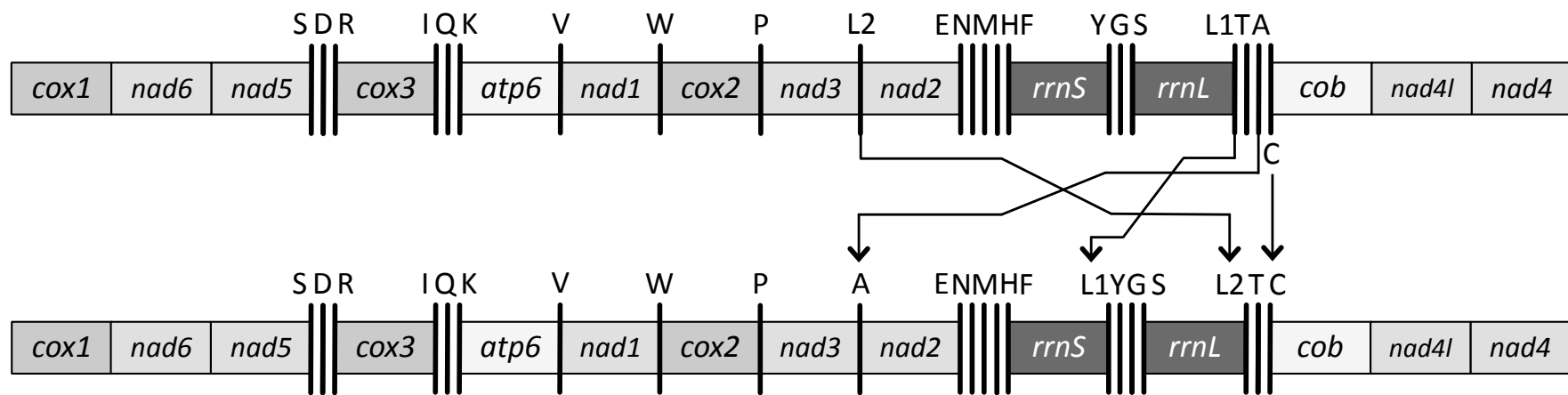

Proposed new annotation

Supplement: S3 Fig — (PDF) [file pone.0120081.s003.pdf]

A)

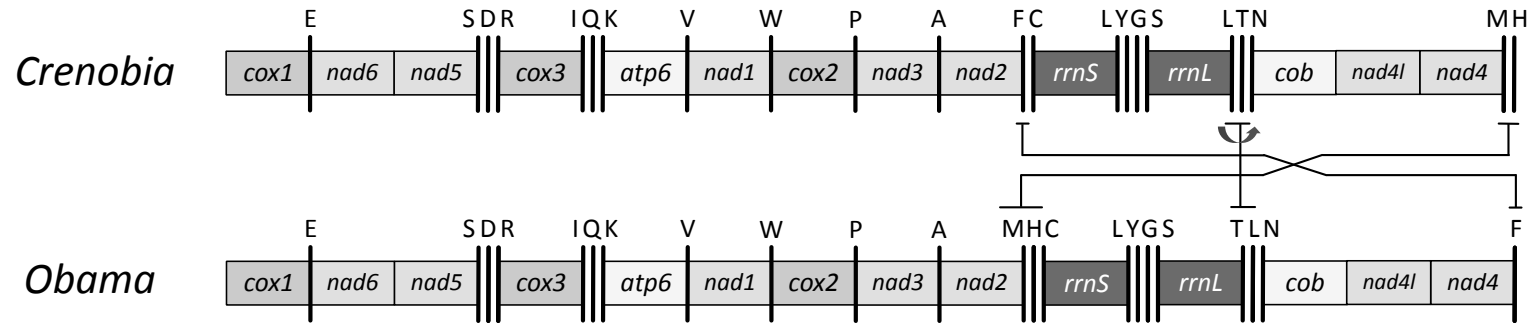

B)

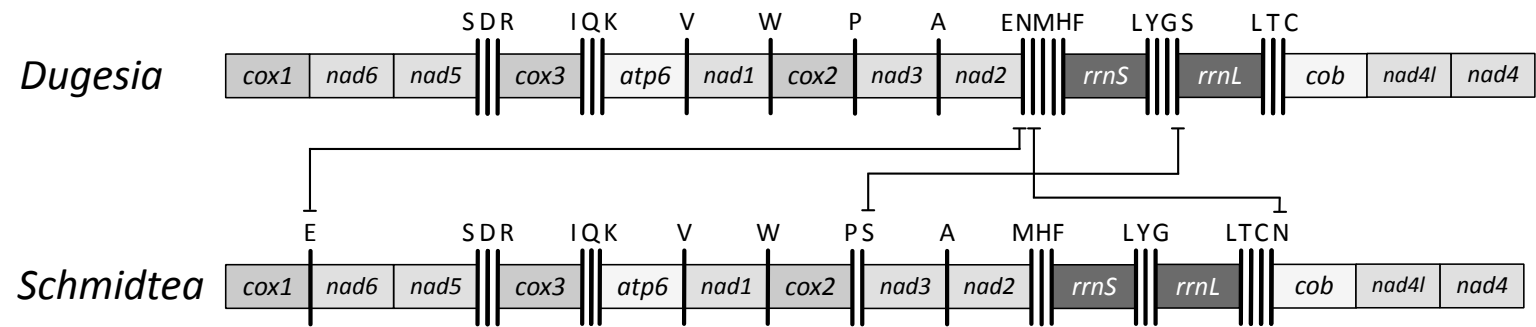

C)

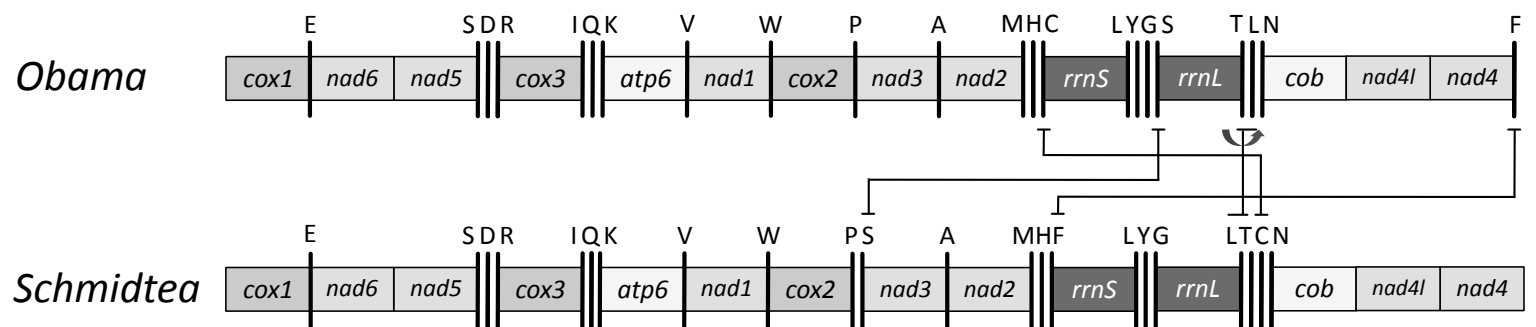

Supplement: S4 Fig — (PDF) [file pone.0120081.s004.pdf]

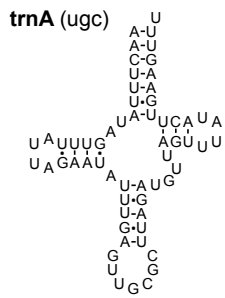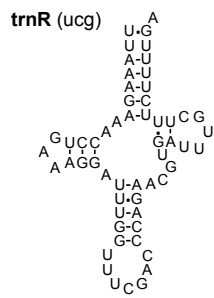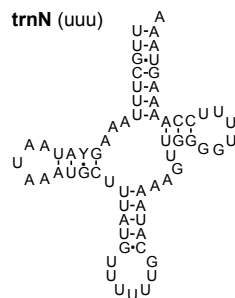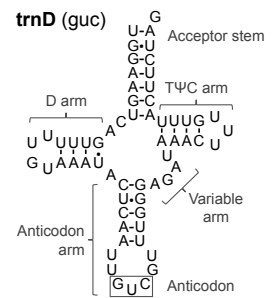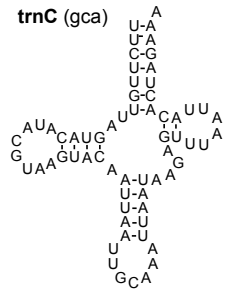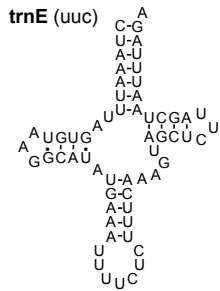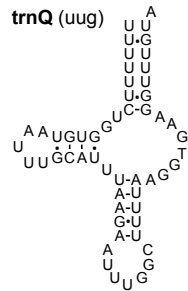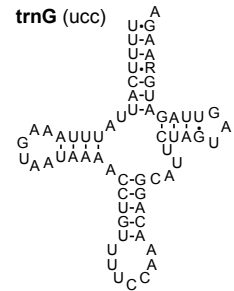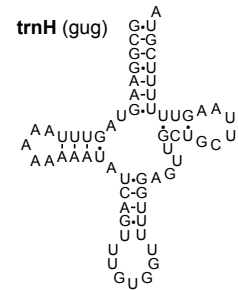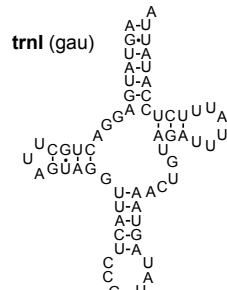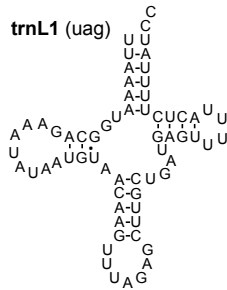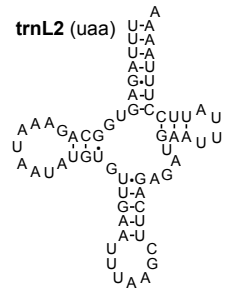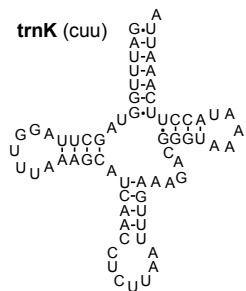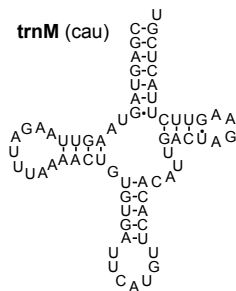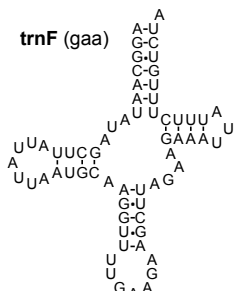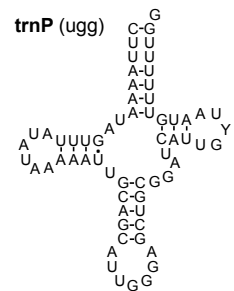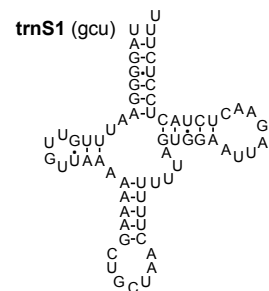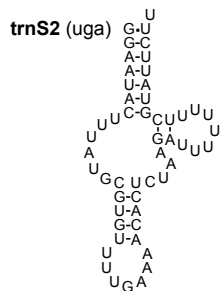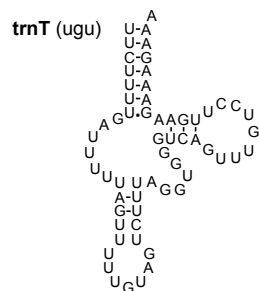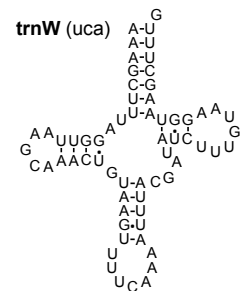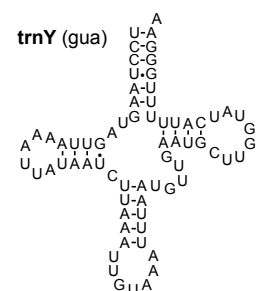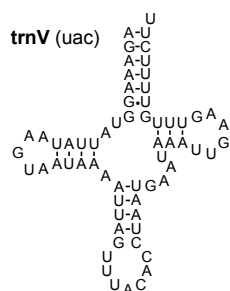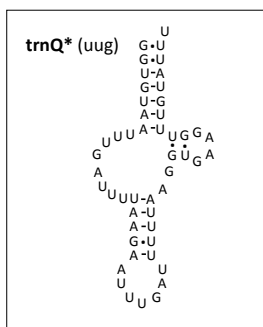

Supplement: S5 Fig — The different tRNA parts are showed on trnD. (PDF) [file pone.0120081.s005.pdf]

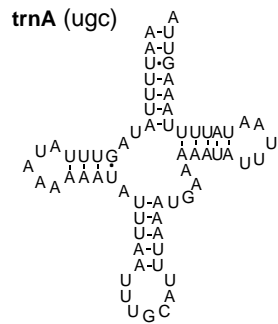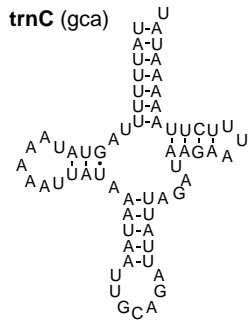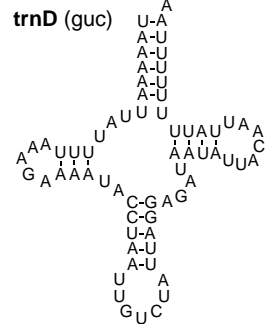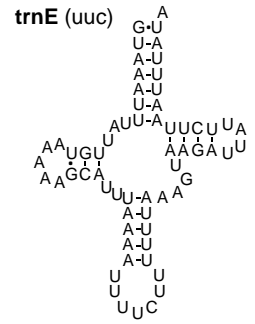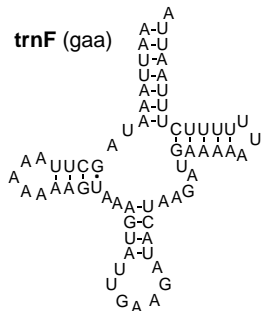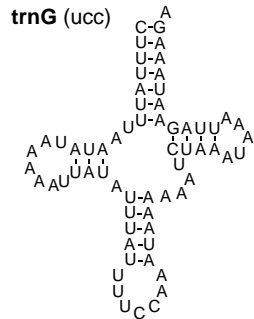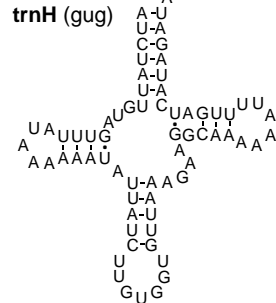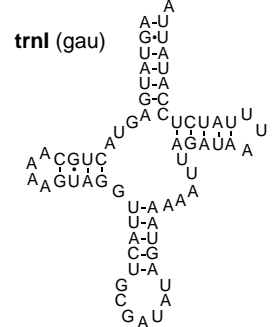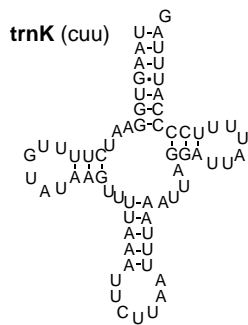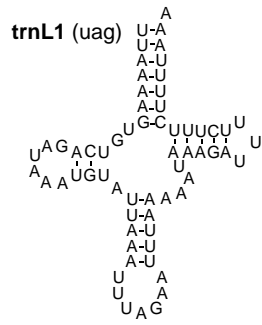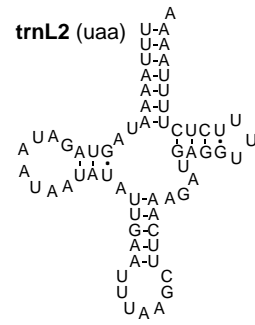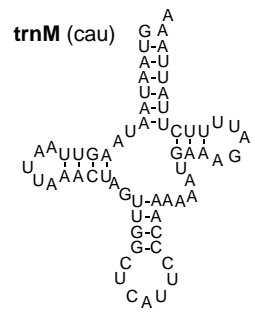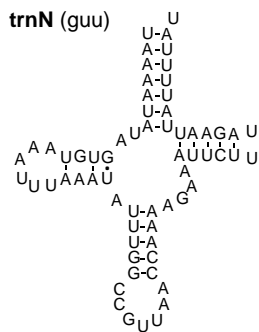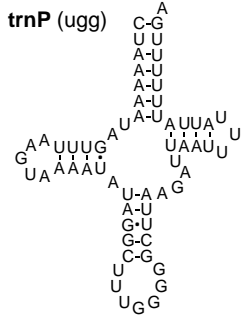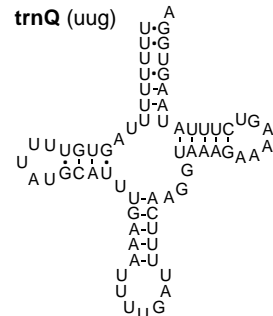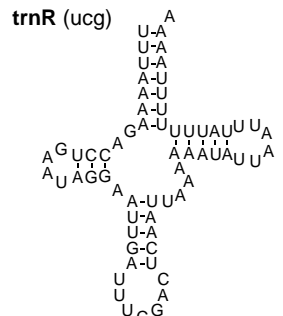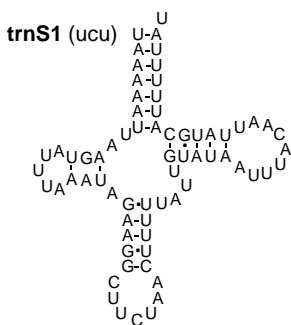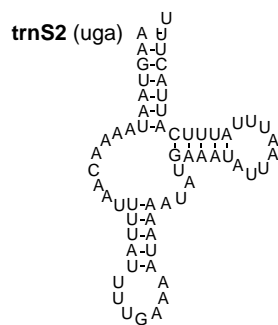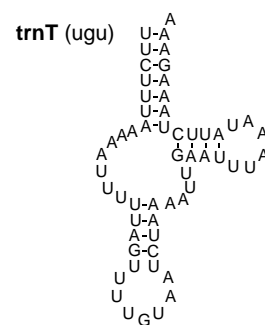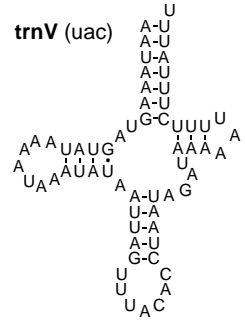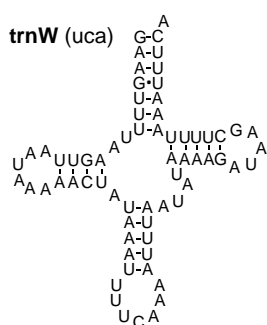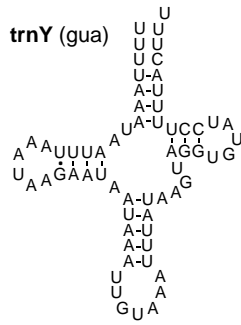

Supplement: S6 Fig — (PDF) [file pone.0120081.s006.pdf]

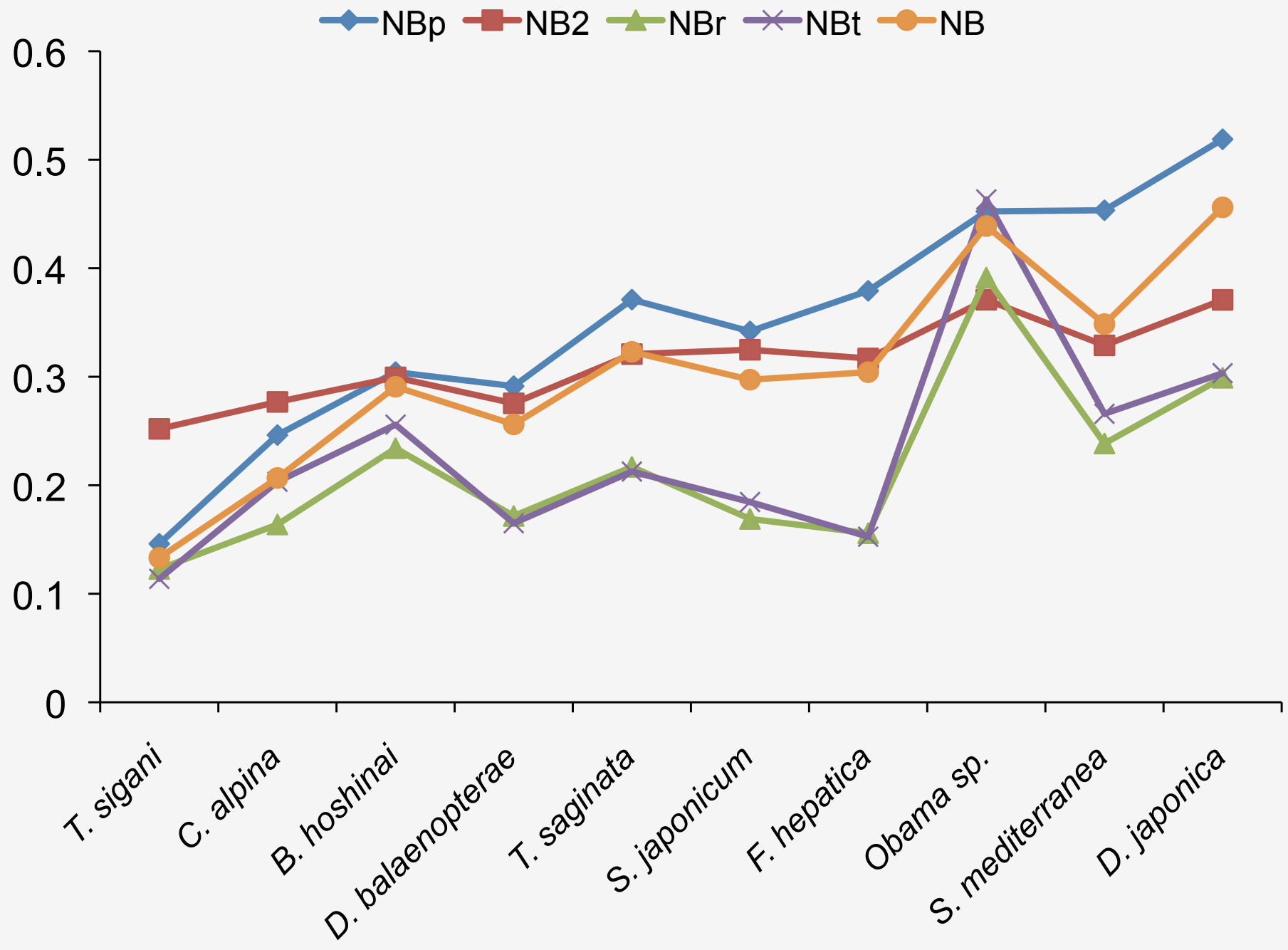

Supplement: S8 Fig — (PDF) [file pone.0120081.s008.pdf]

sAT

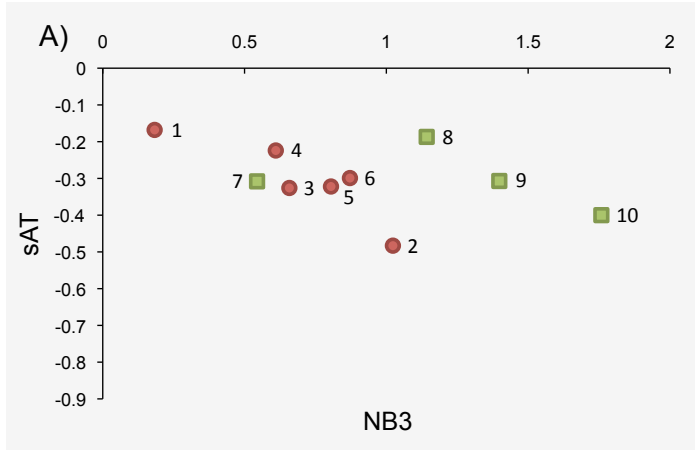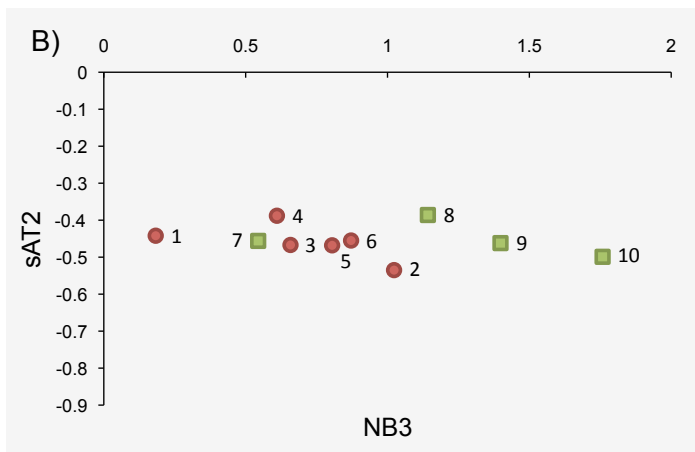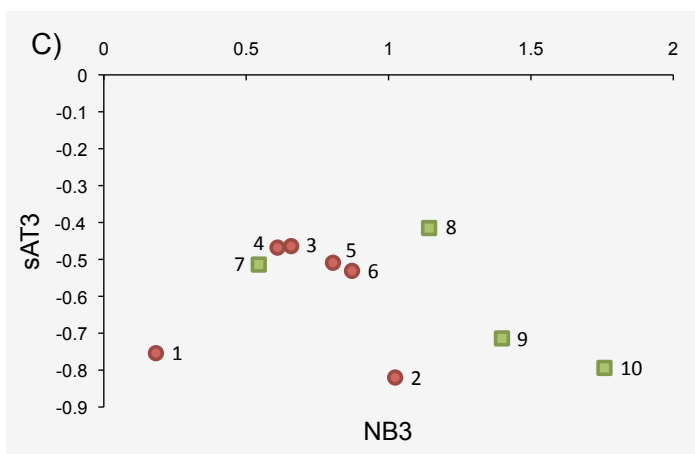

sGC

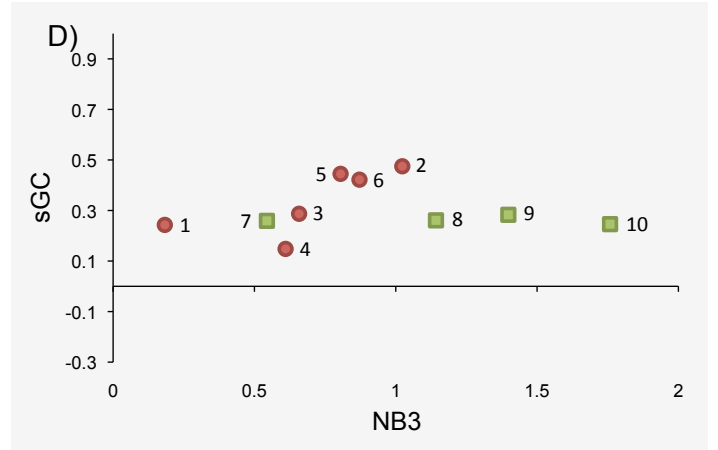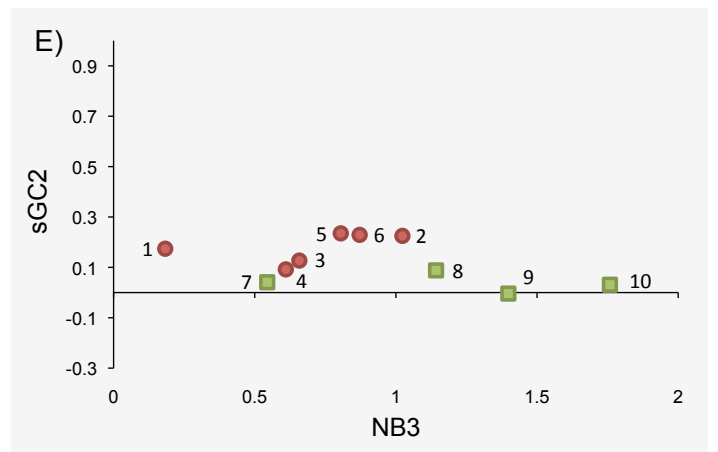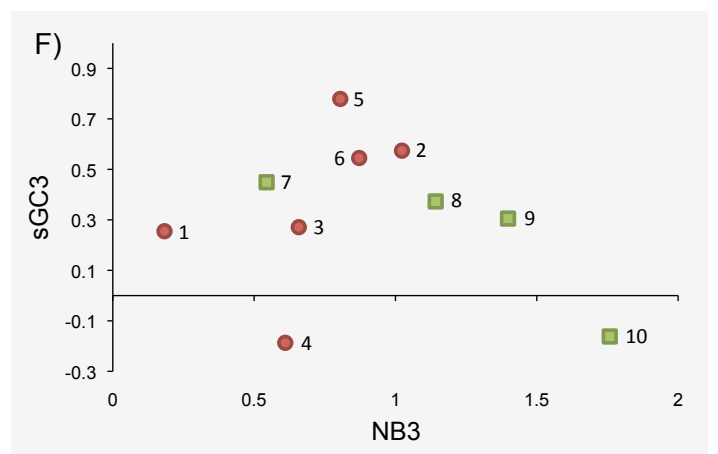

Supplement: S9 Fig — sAT general skew; sAT2, sAT skew at the second positions; sAT3, sAT at the third positions. sGC, general skew; sGC2, sGC skew at the second positions; sGC3, sGC at the third positions. Green squares and red circles indicate free-living and parasitic platyhelminths, respectively. The surveyed species are shown in numbers: 1, T. sigani; 2, F. hepatica; 3, D. balaenopterae; 4, B. hoshinai; 5, T. saginata; 6, S. japonicum; 7, C. alpina; 8, Obama sp.; 9, S. mediterranea; 10, D. japonica. (PDF) [file pone.0120081.s009.pdf]

## sAT

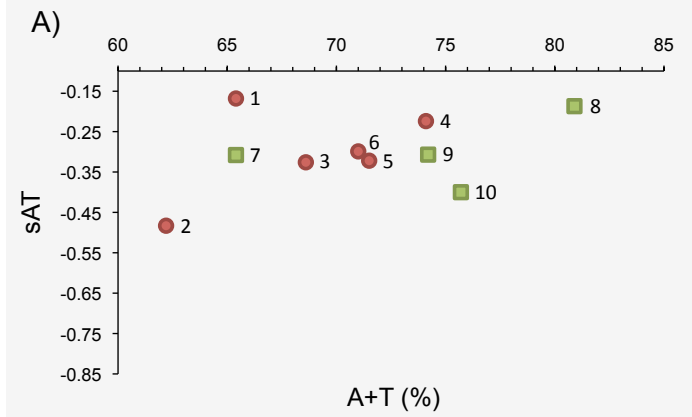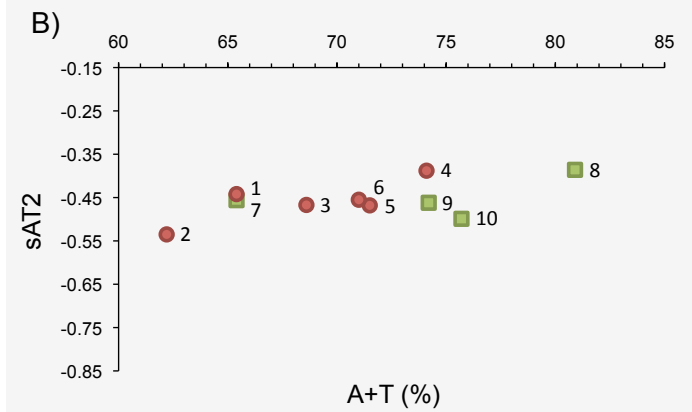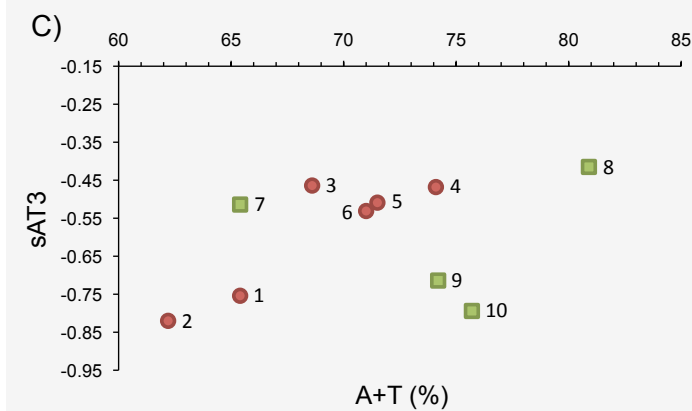

## sGC

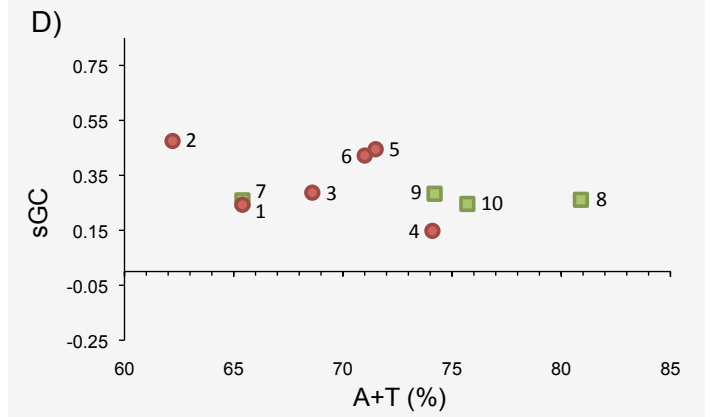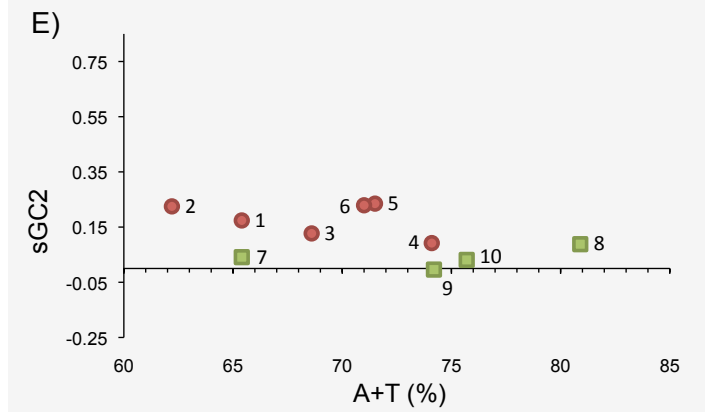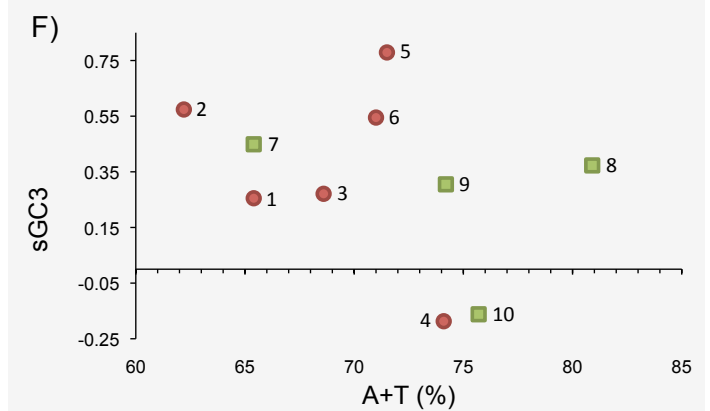

Supplement: S10 Fig — sAT general skew; sAT2, sAT skew at the second positions; sAT3, sAT at the third positions. sGC, general skew; sGC2, sGC skew at the second positions; sGC3, sGC at the third positions. Green squares and red circles indicate free-living and parasitic Platyhelminthes, respectively. The surveyed species are shown in numbers: 1, T. sigani; 2, F. hepatica; 3, D. balaenopterae; 4, B. hoshinai; 5, T. saginata; 6, S. japonicum; 7, C. alpina; 8, Obama sp.; 9, S. mediterranea; 10, D. japonica. (PDF) [file pone.0120081.s010.pdf]

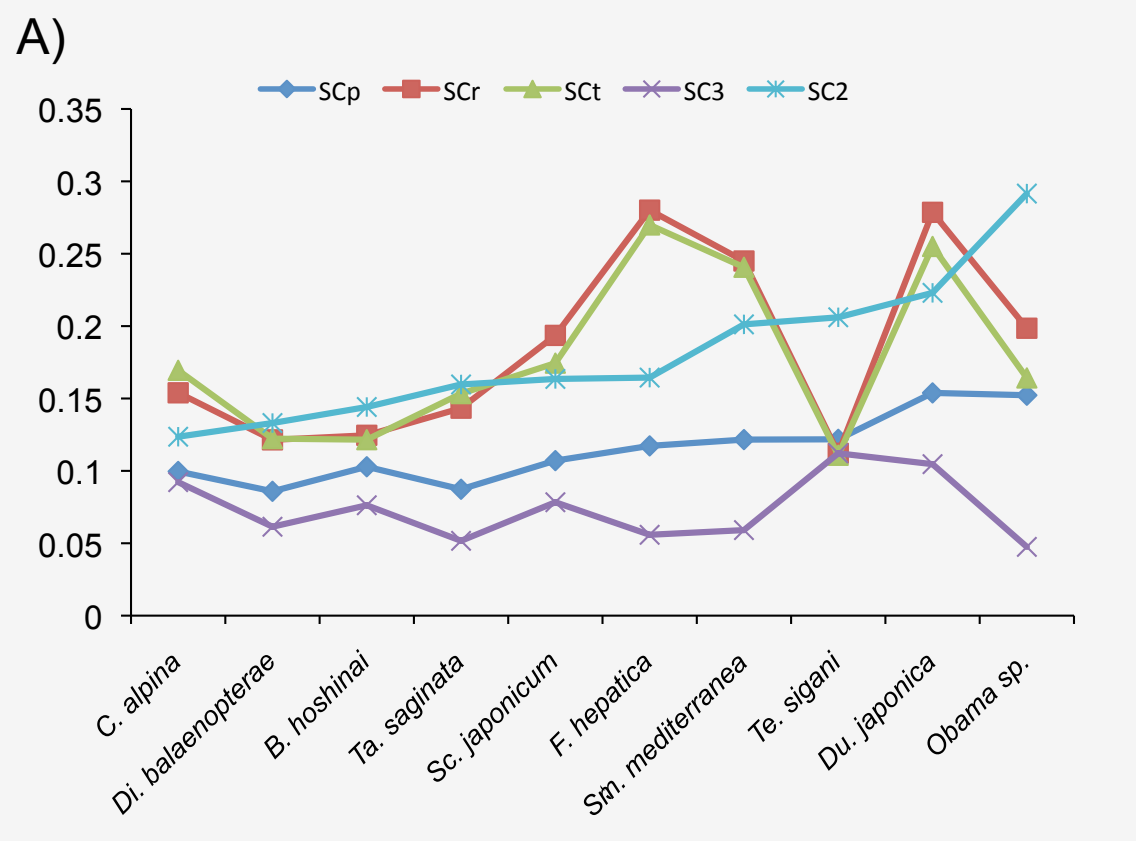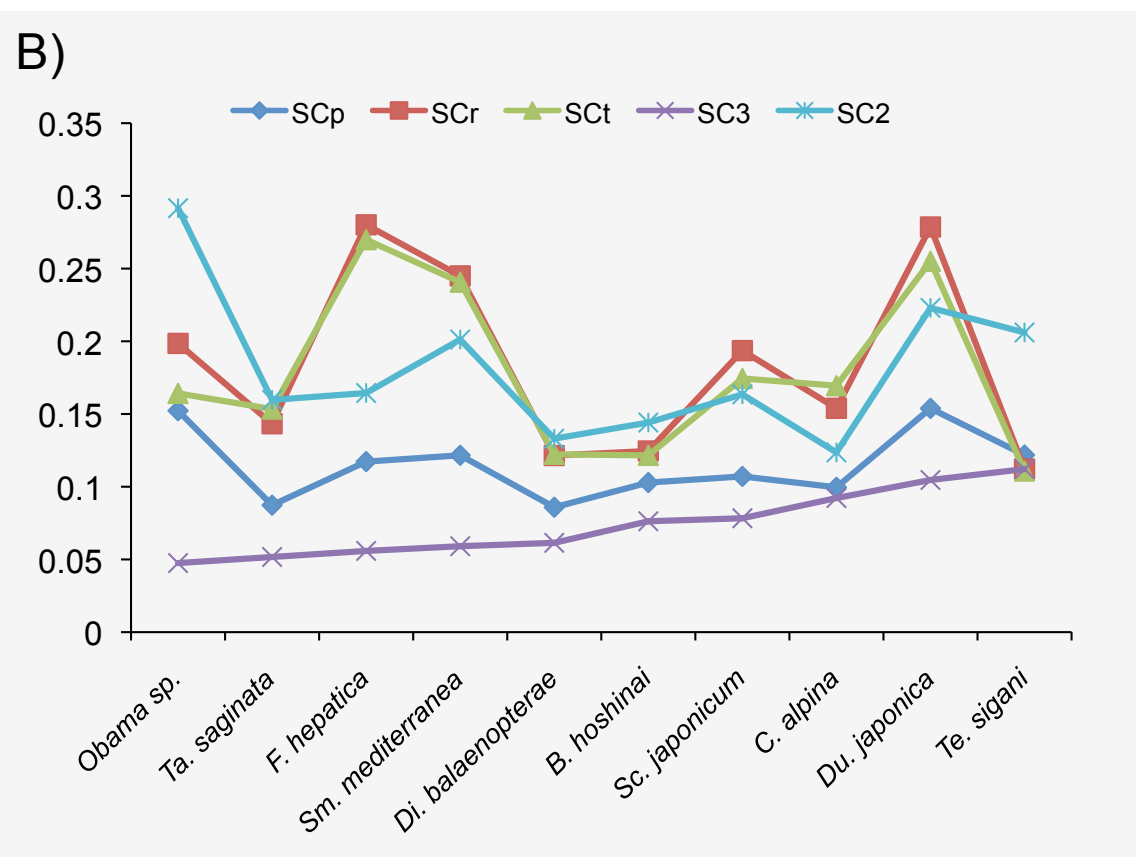

Supplement: S11 Fig — Ordered ascending based on the Chi scales values for A) second positions of the PCG and B) for the third position of four-fold degenerate codons equifrequency. (PDF) [file pone.0120081.s011.pdf]
