# Supplementary material for: Evolutionary Analysis of Mitogenomes from Parasitic and Free-Living Flatworms
Source: PLoS One. 2015 Mar 20;10(3):e0120081. doi: 10.1371/journal.pone.0120081 (PMC4368550; doi:10.1371/journal.pone.0120081)
Supplement: S7 Fig — (PDF) [file pone.0120081.s007.pdf]

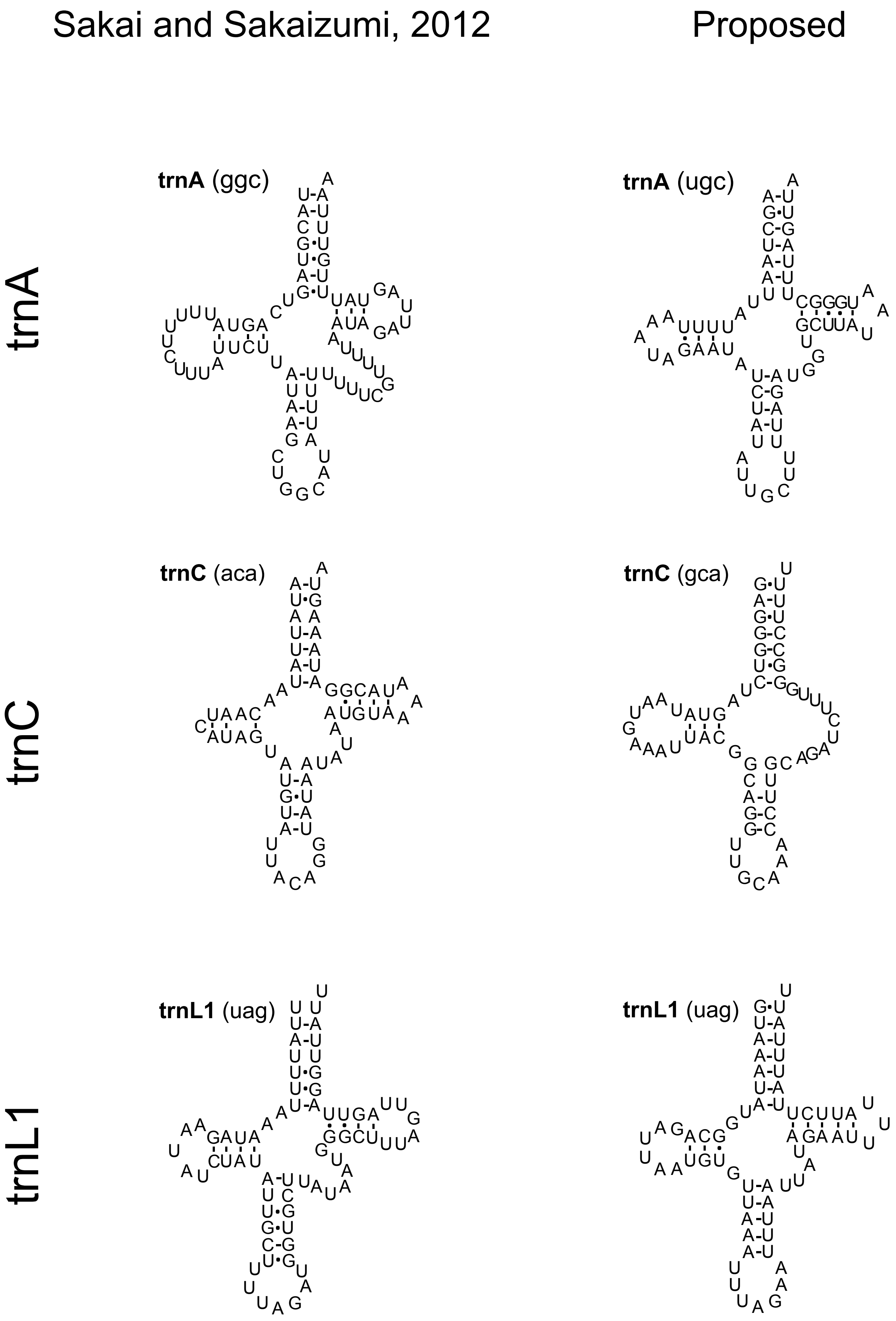

Sakai and Sakaizumi, 2012
Proposed
trnA
trnA (ggc)
trnA (ugc)
trnC
trnC (aca)
trnC (gca)
trnL1
trnL1 (uag)
trnL1 (uag)
