# Supplementary material for: Evolutionary Analysis of Mitogenomes from Parasitic and Free-Living Flatworms
Source: PLoS One. 2015 Mar 20;10(3):e0120081. doi: 10.1371/journal.pone.0120081 (PMC4368550; doi:10.1371/journal.pone.0120081)
Supplement: S1 File — (DOCX) [file pone.0120081.s012.docx]

**Negative results for *Polycelis felina* and *Dugesia subtentaculata***

It was not possible to obtain the mitogenome sequence for two of the species included initially in the study.

In the case of *Polycelis felina* and *Dugesia subtentaculata*, the amount of DNA obtained was within the range needed (2 µg for the first and 500 ng for the second). However, the read length of *P. felina* and *D. subtentaculata* were short (N50 of 246 and 146, respectively), and the prospective tBLASTn analyses showed that only a few reads included protein coding gene (PCG) information (of only 4 or 6 protein coding genes, respectively) (See tables below).

Although it was not possible to obtain a complete assembly for the mitogenome of *D. subtentaculata* we have been able to map the reads obtained on the mitogenome of *Dugesia japonica*, and used that information to develop some specific primers for future studies.

**Summary statistics for the 454 sequencing**

|  |  | ***P. felina*** | ***D.subtentaculata*** |
| --- | --- | --- | --- |
| **Total number (raw) of reads** | | 20.249 | 5.060 |
|  | Mb | 2.82 | 0.61 |
|  | N50 (in bp) | 246 | 146 |
|  | Average length (in bp) | 139.26 | 119.82 |
| **Number of removed reads** | |  |  |
|  | *E. coli* genome seq. | 3 | 11 |
|  | Uni Vecdb | 346 | 124 |
|  | Low quality | 132 | 27 |
|  | Length< 50 bp | 4.716 | 1.131 |
|  | | 15.052 | 3.767 |
| **Number of reads used for the assembling** | |  |  |
|  | Mb | 2.58 | 0.54 |
|  | N50 (in bp) | 270 | 161 |

**Summary of tBLASTn hits for raw reads against the mitochondrial proteins of the three parasitic flatworms.**

|  | ***Polycelis felina*** | | | ***Dugesia subtentaculata*** | | |
| --- | --- | --- | --- | --- | --- | --- |
|  | ***Fhep*** | ***Gder*** | ***Tsol*** | ***Fhep*** | ***Gder*** | ***Tsol*** |
| ***cox3*** | 0 | 1 | 0 | 0 | 0 | 0 |
| ***cob*** | 1 | 1 | 1 | 6 | 7 | 7 |
| ***nad4l*** | 0 | 0 | 0 | 0 | 0 | 0 |
| ***nad4*** | 1 | 2 | 1 | 0 | 1 | 1 |
| ***atp6*** | 0 | 0 | 0 | 0 | 0 | 0 |
| ***nad2*** | 0 | 0 | 0 | 0 | 0 | 0 |
| ***nad1*** | 0 | 0 | 0 | 1 | 1 | 3 |
| ***nad3*** | 0 | 0 | 0 | 0 | 0 | 0 |
| ***cox1*** | 1 | 1 | 1 | 7 | 7 | 7 |
| ***cox2*** | 0 | 0 | 0 | 6 | 6 | 6 |
| ***nad6*** | 0 | 0 | 0 | 0 | 0 | 0 |
| ***nad5*** | 5 | 4 | 4 | 1 | 1 | 1 |

*Fhep*, *Fasciola hepatica*; *Gder*, *Gyrodactylus derjavinoides*; *Tsol*, *Taenia solium*.
