# Supplementary material for: Evolutionary Analysis of Mitogenomes from Parasitic and Free-Living Flatworms
Source: PLoS One. 2015 Mar 20;10(3):e0120081. doi: 10.1371/journal.pone.0120081 (PMC4368550; doi:10.1371/journal.pone.0120081)
Supplement: S1 Tables — Table A. Locality and habitat information on species collected for this study. Table B. Data of mitochondrial proteins used to conduct the tBLASTx analyses in order to detect whether the mitochondrial genes were present in the 454 sequencing reads. Table C. Primers designed for the reamplification of Crenobia alpina. Table D. Primers designed for the reamplification of Obama sp. Table E. Summary statistics for the 454 sequencing. Table F. Summary of tBLASTn hits for raw reads against the mitochondrial proteins of the three parasitic flatworms. Table G. Annotation table for the mitochondrial genome of C. alpina. Table H. Annotation table for the mitochondrial genome of Obama sp. Table I. Annotation table for the mitochondrial genome of S. mediterranea. (DOCX) [file pone.0120081.s013.docx]

| **Species** | **Triclad family** | **Locality** | **Habitat** | **Coordinates** | **Collectors (collection date)** |
| --- | --- | --- | --- | --- | --- |
| *Crenobia alpina* (Dana, 1766) | Planariidae | Rasos de Peguera, Catalonia | Spring | 42° 8'10.77"N 1°45'48.72"E | M. Riutort, E. Solà, M. Álvarez-Presas, L. Leria (02/11/2010) |
| *Polycelis felina* (Dalyell, 1844) | Planariidae | Viladrau, Catalonia | Spring | 41°50'37.82"N 2°23'40.40"E | M. Riutort, E. Mateos, M. Álvarez-Presas (26/10/2010) |
| *Dugesia subtentaculata* (Draparnaud, 1801) | Dugesiidae | Santa Fe del Montseny, Catalonia | River | 41°46'22.23"N 2°28'0.16"E | M. Riutort, E. Solà, M. Álvarez-Presas (22/10/2010) |
| *Obama* sp. Álvarez-Presas *et al.*, 2014 | Geoplanidae | Torruella de Fluvià, Catalonia | Garden | 42°10'29.21"N 3° 2'41.14"E | M. Riutort (12/10/2010) |

**Table A.** Locality and habitat information on species collected for this study.

**Table B.** Data of mitochondrial proteins used to conduct the tBLASTx analyses in order to detect whether the mitochondrial genes were present in the 454 sequencing reads.

|  |  | ***Fasciola hepatica*** | ***Gyrodactylus derjavinoides*** | ***Taenia solium*** |
| --- | --- | --- | --- | --- |
| **Protein name** | **Locus** | **Protein product** | **Protein product** | **Protein product** |
| cytochrome c oxidase subunit III | *cox3* | NP_0666217.2 | YP_001974722.1 | NP_659225.1 |
| cytochrome b | *cob* | NP_0662218.2 | YP_001974723.1 | NP_659226.1 |
| NADH dehydrogenase subunit 4L | *nad4l* | NP_0662219.1 | YP_001974724.1 | NP_659227.1 |
| NADH dehydrogenase subunit 4 | *nad4* | NP_066220.2 | YP_001974725.1 | NP_659228.1 |
| ATP synthase F0 subunit 6 | *atp6* | NP_066221.2 | YP_001974726.1 | NP_659229.1 |
| NADH dehydrogenase subunit 2 | *nad2* | NP_066222.2 | YP_001974727.1 | NP_659230.1 |
| NADH dehydrogenase subunit 1 | *nad1* | NP_066223.2 | YP_001974728.1 | NP_659231.1 |
| NADH dehydrogenase subunit 3 | *nad3* | NP_066224.2 | YP_001974729.1 | NP_659232.1 |
| cytochrome c oxidase subunit I | *cox1* | NP_066225.2 | YP_001974730.1 | NP_659233.1 |
| cytochrome c oxidase subunit II | *cox2* | NP_066226.2 | YP_001974731.1 | NP_659234.1 |
| NADH dehydrogenase subunit 6 | *nad6* | NP_066227.2 | YP_001974732.1 | NP_659235.1 |
| NADH dehydrogenase subunit 5 | *nad5* | NP_066228.2 | YP_001974733.1 | NP_659224.1 |

**Table C.** Primers designed for the reamplification of *Crenobia alpina*.

| **Name** |  | **Sequence 5'-3'** | **Annealing T (ºC)** | **Genes** |
| --- | --- | --- | --- | --- |
| Tinc | F | GATTGCTACGGGTTTGG | 49 | *cob*^a^; *nad4l*^a^ |
| Gana | R | CACATTCCTCTTATCCC | 42.2 |  |
| Joan | F | GTGAAGGTTTTGGGG | 44.1 | *nad4l*; *nad4*^a^ |
| Dora | R | CCCTTCCAACACTCC | 44 |  |
| Ste | F | GGTTGGTGTTTTCGG | 45.3 | *nad4*^a^; *trnM* (cau); trnH (gug) |
| Phen | R | CAACCAAAACCGCCAAG | 42.8 |  |
| Dar | F | GGGTTGAAAGATGTGCGG | 54.2 | *cox1*^a^ |
| Win | R | CCAAAACCGCCAATC | 48.2 |  |
| Ice | F | GTATTTCTTTGGGGTTGG | 46.8 | *cox1*^a^ |
| Age | R | CTCCCCAGCCATTCC | 50.1 |  |
| Dino | F | GGGTTCTTTATTGTCTTTGCTTAGCG | 47.2 | *cox1*^a^; *trnE* (uuc); |
|  |  |  |  | *nad6*; *nad5*^a^ |
| Saure | R | CAGCGAGCATTGTGAATAGTCC | 45.7 |  |
| Rap | F | CCCAGTATCCTTTTTC | 39.4 | *nad5*^a^ |
| Tor | R | ACAAGCATAAAGTATTCCC | 43.2 |  |
| Chi | F | TCTTTTGTCCGCTTCTG | 47.4 | *nad5*^a^; *trnS2* (ugc); *trnD* (guc); *trnR* (ucg); *cox3*; *trnI* (gau); *trnQ* *(uug)*^a^ |
| Cago | R | CCGAAATACAAACCTTC | 42.6 |  |
| Angi | F | CACTCTTCTTTGCGTTG | 45.2 | *cox3*^a^; *trnI* *(gau)*; trnQ (uug); trnK (cuu); atp6; trnV (uac) |
| Laia | R | CAACAACCCCCAAAAC | 47 |  |
| Ptero | F | GGGTGTATGTGGACTTTTG | 47.8 | *atp6*^a^; *trnV* (uac); *nad1*; *trnW* (uca); *cox2* |
| Dactil | R | GAAACAATCTAACTGCTCC | 43.7 |  |
| Wil | F | CTTTGCTTGGTCCATTG | 47.6 | *nad1*^a^; |
| Son | R | CCACGACGCTTCTCCTC | 52.3 |  |
| Eva | F | GAGTGTGGTTTTGATGG | 44.2 | *nad3*^a^; *trnA* (ugc); *nad2*^a^ |
| Ona | R | CCCAGAAAACACAAAGAAAC | 48.7 |  |
| Cholo | F | GTGTTCTCTTATGTCTCC | 38.3 | *nad2*^a^; *trnF* (gaa); RR^a^ |
| Epus | R | CCCCTTATTTTCCAC | 40.1 |  |
| Trilo2 | F | GGGAAATAGAAGGAGGG | 45.9 |  |
| Bite | R | CTAAGGGGAGGGTTGGG | 51.9 |  |
| Brady | F | GTTGAAGAATGAGACTG | 37.1 | *trnC* (gca); *rrnS*^a^ |
| Pus | R | GAATAGTGACGGGCGGTG | 54.2 |  |
| New | F | GAAAGATAGATAGAGGGG | 39.5 | *rrnS*^a^; *trnL1* (uag); trnY (gua); trnG (ucc); rrnL^a^ |
| York | R | CCTTCATATTAAACCCGTTC | 53.7 |  |
| Artro | F | GTATCCCCTGCTCGTTG | 49.2 | *rrnL*^a^; *trnL2* (uaa); trnT (ugu); trnN (guu); cob^a^ |
| Pode | R | CAACCCTCTTCCCCAC | 52.5 |  |

^a^ The gene is covered partially by the primers.

**Table D.** Primers designed for the reamplification of *Obama* sp.

| **Name** |  | **Sequence 5'-3'** | **Annealing T (ºC)** | **Genes** |
| --- | --- | --- | --- | --- |
| Grand | F | GAAAGKKAGGAGGTG | 40.8 | *cob*^a^; *nad4l*^a^; *nad4*^a^ |
| Jete | R | CTTTAHATCAWACTGAC | 37.1 |  |
| Kete | F | CATGGTTTTTGTTCTTC | 50.6 | *nad4*^a^; *trnF* (gaa); *cox1*^a^ |
| Peten | R | CCAAAACCACCAATC | 51.9 |  |
| Ni | F | GGTTTTATTGTTTGAGC | 49.9 | *cox1*^a^; *trnE* (uuc); *nad6*; *nad5*^a^ |
| Jinsky | R | CCATCYCAACCAAAC | 48.6 |  |
| Pau | F | CTGCTTTAGTTCATTC | 44.6 | *nad5*^a^; *trnS2 (uga)*; trnD (guc); trnR (ucg); cox3^a^ |
| Lova | R | GWAAACCATGAAAACCAG | 50.2 |  |
| Bat | F | GCAGYTTGATATTGRC | 46.4 | *cox3*^a^; *trnI* (gau); trnQ (uug); trnK (cuu); atp6; trnV (uac); nad1^a^ |
| Man | R | CGAATCTGBATATABCTC | 40.4 |  |
| Porde | F | GGTTCTTTDGARTTTGC | 48.7 | *nad1*^a^; *trnW* (uca); *cox2*; *trnP* (ugg); *nad3*^a^ |
| Bra | R | GMARACGAGAMATATAC | 23.7 |  |
| Enri | F | GARGAATTRCGTHGTGG | 39.2 | *nad3*^a^; *trnA* (ugc); *nad2*^a^ |
| Kito | R | GAAGATYCAARCC | 29 |  |
| Ene | F | GGYTTGRTCTTC | 27 | *nad2*^a^; *trnM* (cau); *trnH* (gug); *trnC* (gca); *rrnS*^a^ |
| Sim | R | GYTGCTGGCACYC | 35 |  |
| Valen | F | GTTAGTGTACGGTTG | 42.2 | *rrnS*^a^; *trnL1* (uag); trnY (gua); trnG (ucc); trnS1 (ucu); rrnLa |
| Tin | R | CGGTCTAAACTCAAATC | 49.8 |  |
| Demi | F | CGAAAAGACCCTACAG | 50.7 | *rrnL*^a^; *trnT* (ugu); trnL2 (uaa); trnN (guu); RR; cob^a^ |
| Plie | R | GTAATAACAGTAGCDCC | 42.9 |  |

^a^ The gene is covered partially by the primers.

**Table E.** Summary statistics for the 454 sequencing.

|  |  | ***Crenobia alpina*** | ***Obama* sp.** |
| --- | --- | --- | --- |
| **Total number (raw) of reads** | | 31.282 | 11.671 |
|  | Mb | 6.19 | 3.77 |
|  | N50 (in bp) | 377 | 427 |
|  | Average length (in bp) | 197.96 | 323.22 |
| **Number of removed reads** | |  |  |
|  | *E. coli* genome seq. | 3 | 221 |
|  | Uni Vecdb | 545 | 102 |
|  | Low quality | 205 | 114 |
|  | Length< 50 bp | 5.399 | 720 |
|  | | 25.130 | 10.614 |
| **Number of reads used for the assembling** | |  |  |
|  | Mb | 5.88 | 3.59 |
|  | N50 (in bp) | 382 | 425 |

**Table F.** Summary of tBLASTn hits for raw reads against the mitochondrial proteins of the three parasitic flatworms.

|  | ***Crenobia alpina*** | | | ***Obama* sp.** | | |
| --- | --- | --- | --- | --- | --- | --- |
|  | ***Fhep*** | ***Gder*** | ***Tsol*** | ***Fhep*** | ***Gder*** | ***Tsol*** |
| ***cox3*** | 2 | 0 | 0 | 2 | 0 | 0 |
| ***cob*** | 55 | 67 | 62 | 58 | 64 | 64 |
| ***nad4l*** | 0 | 0 | 0 | 0 | 0 | 0 |
| ***nad4*** | 27 | 16 | 15 | 11 | 17 | 10 |
| ***atp6*** | 0 | 2 | 1 | 1 | 2 | 1 |
| ***nad2*** | 0 | 0 | 0 | 0 | 0 | 0 |
| ***nad1*** | 48 | 45 | 52 | 41 | 44 | 43 |
| ***nad3*** | 0 | 0 | 8 | 0 | 0 | 0 |
| ***cox1*** | 126 | 132 | 117 | 176 | 191 | 187 |
| ***cox2*** | 43 | 47 | 45 | 30 | 33 | 29 |
| ***nad6*** | 0 | 0 | 0 | 0 | 0 | 0 |
| ***nad5*** | 51 | 49 | 48 | 37 | 40 | 41 |

*Fhep*, *Fasciola hepatica*; *Gder*, *Gyrodactylus derjavinoides*; *Tsol*, *Taenia solium*.

**Table G.** Annotation table for the mitochondrial genome of *C. alpina*.

| **Gene** | **Start** | **End** | **Start codon** | **Stop Codon** | **Size (bp)** |
| --- | --- | --- | --- | --- | --- |
| *cob* | 1 | 1113 | ATG | TAG | 1113 |
| *nad4l* | 1157 | 1390 | TTG | TAG | 234 |
| *nad4* | 1359 | 2714 | ATG | TAA | 1356 |
| *trnM* (cau) | 2722 | 2789 |  |  | 68 |
| *trnH* (gug) | 2925 | 2988 |  |  | 64 |
| *cox1* | 3359 | 5113 | ATG | TAG | 1755 |
| *trnE* (uuc) | 5117 | 5177 |  |  | 61 |
| *nad6* | 5179 | 5664 | ATG | TAA | 486 |
| *nad5* | 5665 | 7314 | ATG | TAG | 1650 |
| *trnS2* (uga) | 7427 | 7479 |  |  | 53 |
| *trnD* (guc) | 7483 | 7544 |  |  | 62 |
| *trnR* (ucg) | 7545 | 7601 |  |  | 57 |
| *cox3* | 7633 | 8430 | ATG | TAG | 798 |
| *trnI* (gau) | 8429 | 8492 |  |  | 64 |
| *trnQ* (uug) | 8493 | 8547 |  |  | 55 |
| *trnK* (cuu) | 8553 | 8620 |  |  | 68 |
| *atp6* | 8624 | 9277 | TTG | TAA | 654 |
| *trnV* (uac) | 9280 | 9344 |  |  | 65 |
| *nad1* | 9411 | 10235 | ATG | TAA | 825 |
| *trnW* (uca) | 10234 | 10298 |  |  | 65 |
| *cox2* | 10302 | 11021 | ATG | TAR | 720 |
| *trnP* (ugg) | 11030 | 11093 |  |  | 64 |
| *nad3* | 11118 | 11450 | TTG | TAA | 333 |
| *trnA* (ugc) | 11449 | 11509 |  |  | 61 |
| *nad2* | 11515 | 12486 | TTA | TAG | 972 |
| *trnF* (gaa) | 12485 | 12550 |  |  | 66 |
| *trnC* (gca) | 14582 | 14646 |  |  | 65 |
| *rrnS* | 14676 | 15308 |  |  | 633 |
| *trnL1* (uag) | 15310 | 15374 |  |  | 65 |
| *trnY* (gua) | 15372 | 15441 |  |  | 70 |
| *trnG* (ucc) | 15444 | 15507 |  |  | 64 |
| *trnS1 (ucu)* | 15509 | 15575 |  |  | 67 |
| *rrnL* | 15637 | 16494 |  |  | 858 |
| *trnL2* (uaa) | 16656 | 16719 |  |  | 64 |
| *trnT* (ugu) | 16720 | 16777 |  |  | 64 |
| *trnN* (guu) | 16784 | 16852 |  |  | 69 |

**Table H.** Annotation table for the mitochondrial genome of *Obama* sp.

| **Gene** | **Start** | **End** | **Start Codon** | **Stop Codon** | **Size (bp)** |
| --- | --- | --- | --- | --- | --- |
| *cob* | 1 | 1182 | ATG | TAG | 1182 |
| *nad4l* | 1166 | 1399 | TTA | TAG | 234 |
| *nad4* | 1368 | 2789 | ATG | TAA | 1422 |
| *TrnF* (gaa) | 2789 | 2853 |  |  | 65 |
| *cox1* | 2854 | 4590 | GTG | TAA | 1737 |
| *trnE* (uuc) | 4593 | 4656 |  |  | 64 |
| *nad6* | 4658 | 5134 | TTG | TAA | 477 |
| *nad5* | 5138 | 6772 | TTG | TAA | 1635 |
| *trnS2* (uga) | 6773 | 6834 |  |  | 62 |
| *trnD* (guc) | 6835 | 6901 |  |  | 67 |
| *trnR* (ucg) | 6897 | 6961 |  |  | 65 |
| *cox3* | 6962 | 7753 | ATG | TAA | 792 |
| *trnI* (gau) | 7754 | 7819 |  |  | 66 |
| *trnQ* (uug) | 7821 | 7887 |  |  | 67 |
| *trnK* (cuu) | 7880 | 7944 |  |  | 65 |
| *atp6* | 7945 | 8619 | TTG | TAA | 675 |
| *trnV* (uac) | 8619 | 8681 |  |  | 63 |
| *nad1* | 8684 | 9577 | ATG | TAA | 894 |
| *trnW* (uca) | 9580 | 9647 |  |  | 68 |
| *cox2* | 9648 | 10427 | TTG | TAA | 780 |
| *trnP* (ugg) | 10429 | 10491 |  |  | 63 |
| *nad3* | 10510 | 10851 | TTG | TAA | 342 |
| *trnA* (ugc) | 10852 | 10918 |  |  | 67 |
| *nad2* | 10925 | 11923 | ATG | TAA | 999 |
| *trnM* (cau) | 12076 | 12138 |  |  | 63 |
| *trnH* (gug) | 12144 | 12212 |  |  | 69 |
| *trnC* (gca) | 12213 | 12275 |  |  | 63 |
| *rrnS* | 12311 | 12972 |  |  | 662 |
| *trnL1* (uag) | 12973 | 13036 |  |  | 64 |
| *trnY* (gua) | 13037 | 13099 |  |  | 63 |
| *trnG* (ucc) | 13096 | 13160 |  |  | 65 |
| *trnS1* (ucu) | 13161 | 13229 |  |  | 69 |
| *rrnL* | 13259 | 14178 |  |  | 920 |
| *trnT* (ugu) | 14181 | 14237 |  |  | 57 |
| *trnL2* (uaa) | 14238 | 14301 |  |  | 64 |
| *trnN* (guu) | 14302 | 14366 |  |  | 65 |

**Table I.** Annotation table for the mitochondrial genome of *S. mediterranea*.

| **Gene** | **Start** | **End** | **Start Codon** | **Stop Codon** | **Size (bp)** |
| --- | --- | --- | --- | --- | --- |
| *cob* | 1 | 1101 | TAT* | TAG | 1101 |
| *nad4l* | 1068 | 1361 | ATG | TAG | 294 |
| *nad4* | 1312 | 2688 | ATG | TAG | 1377 |
| *cox1* | 3391 | 5023 | TAT* | TAA | 1633 |
| *trnE* (uuc) | 5024 | 5085 |  |  | 62 |
| *nad6* | 5096 | 5542 | ATG | TAG | 447 |
| *nad5* | 5539 | 7134 | TTA* | TAA | 1596 |
| *trnS2* (uga) | 7138 | 7196 |  |  | 59 |
| *trnD* (guc) | 7197 | 7258 |  |  | 62 |
| *trnR* (ucg) | 7258 | 7319 |  |  | 62 |
| *cox3* | 7317 | 8144 | TAT* | TAA | 828 |
| *trnI* (gau) | 8138 | 8205 |  |  | 68 |
| *trnQ* (uug) | 8204 | 8268 |  |  | 65 |
| *trnK* (cuu) | 8268 | 8331 |  |  | 64 |
| *atp6* | 8334 | 8969 | ATG | TAG | 636 |
| *trnV* (uac) | 8971 | 9033 |  |  | 63 |
| *nad1* | 9030 | 9920 | ATG | TAA | 891 |
| *trnW* (uca) | 9924 | 9988 |  |  | 65 |
| *cox2* | 9989 | 10867 | TTG* | TAA | 879 |
| *trnP* (ugg) | 10971 | 11041 |  |  | 71 |
| *trnS1* (ucu) | 11071 | 11123 |  |  | 53 |
| *nad3* | 11126 | 11395 | TTG* | TAG | 270 |
| *trnA* (ugc) | 11400 | 11468 |  |  | 69 |
| *nad2* | 11547 | 12416 | ATG | TAA | 870 |
| *trnM* (cau) | 24128 | 24190 |  |  | 63 |
| *trnH* (gug) | 24193 | 24259 |  |  | 67 |
| *trnF* (gaa) | 24263 | 24328 |  |  | 66 |
| *rrnS* | 24330 | 25036 |  |  | 707 |
| *trnL1* (uag) | 25038 | 25100 |  |  | 63 |
| *trnY* (gua) | 25106 | 25171 |  |  | 66 |
| *trnG* (ucc) | 25177 | 25245 |  |  | 69 |
| *rrnL* | 25256 | 26160 |  |  | 905 |
| *trnL2* (uaa) | 26161 | 26223 |  |  | 63 |
| *trnT* (ugu) | 26224 | 26277 |  |  | 54 |
| *trnC* (gca) | 26292 | 26351 |  |  | 60 |
| *trnN* (guu) | 26361 | 26424 |  |  | 64 |
